# Human‐supervised LLM triage of pig butchering complaints: A validation study of multi‐model identifier extraction

**DOI:** 10.1111/1556-4029.70411

**Published:** 2026-07-22

**Authors:** Sanghyeob Ko, Jumi Kim, Jin Young Jang, Gibum Kim

**Affiliations:** ^1^ Department of Forensic Science Sungkyunkwan University Seoul Republic of Korea; ^2^ Korean Police Investigation Academy Asan Republic of Korea; ^3^ Daejeon Provincial Police Agency Daejeon Republic of Korea

**Keywords:** blockchain forensics, cryptocurrency fraud, digital forensic triage, human‐supervised triage, identifier extraction, large language models, multi‐model validation, pig butchering scam

## Abstract

Digital forensic investigations increasingly process unstructured cryptocurrency‐fraud complaints at intake while preserving analyst oversight before evidentiary or downstream investigative use. This validation study evaluates whether human‐supervised multi‐model LLM extraction can recover triage‐relevant identifiers from California DFPI pig butchering complaints (2022–2024, *N* = 273). We introduce a four‐tier, information‐based forensic taxonomy that classifies cases by the presence of traceable identifiers—cryptocurrency wallet addresses, fraud‐related platform URLs, and reported loss amounts—rather than by narrative length. Three commercial LLMs (Claude Sonnet 4.5, GPT‐5.2, Gemini 3 Pro) were evaluated against adjudicated ground truth annotated by two independent raters (Cohen's *κ* = 0.969–0.988). The taxonomy classified 96.7% of cases (264/273) as actionable, with 9.2% containing wallet addresses that support direct blockchain tracing. LLM extraction achieved F1 = 1.000 for wallet addresses, F1 = 0.911–0.918 for URLs, and F1 = 0.865–0.904 for loss amounts; lexical regex and pre‐trained spaCy NER baselines achieved F1 ≤0.381 for loss extraction, where context‐dependent interpretation is required. The pipeline routed 82 of the 264 actionable cases (31.1%) to prioritized human review based on cross‐model URL or loss‐amount discrepancies, functioning as a quality‐control mechanism rather than an output‐aggregation ensemble. Pipeline‐stage extraction completed in 74 min at approximately $15 USD; this efficiency is reported separately from the human‐review queue that the framework mandates by design. Because commercial API processing involves data‐sovereignty and CJIS‐equivalent compliance considerations, we position the protocol as triage support within analyst‐mediated workflows. We identify local deployment and pre‐transmission anonymization as priorities for future operational validation.

**Highlights**

- A four‐tier information‐based forensic taxonomy classified 96.7% of cases by identifiers.
- Tri‐model LLMs recovered fraud identifiers at F1 = 0.865–1.000 across artifact types.
- Cross‐model disagreement directed 31% of cases to prioritized human review as quality control.
- Pipeline extraction took 74 min at $15 USD; flagged cases remained in human review.
- Screenshot cross‐check on 25 cases surfaced image‐only leads as a pilot finding for future work.

## 1 INTRODUCTION

Digital forensic investigations increasingly confront the challenge of processing massive volumes of unstructured victim reports. In cryptocurrency fraud cases, particularly the emerging “pig butchering” scam, investigators manually extract actionable intelligence from lengthy narrative statements containing complex social engineering tactics, fraudulent platform details, and blockchain transaction identifiers. This manual triage process creates significant bottlenecks in resource‐constrained law enforcement agencies, delaying the identification of high‐priority cases that warrant immediate technical investigation.

Pig butchering scams combine emotional manipulation with cryptocurrency investment deception, guiding victims through multi‐stage investment platforms before executing systematic financial extraction [1, 2]. The California Department of Financial Protection and Innovation (DFPI) reported 273 such cases in their public Crypto Scam Tracker, with reported losses exceeding $52 million based on our dataset analysis. The unstructured nature of victim narratives—ranging from brief descriptions to multi‐page accounts—makes systematic pattern identification and resource prioritization extremely challenging for forensic analysts. Based on practitioner input, manual processing of a single case can require approximately 2 h. This limits investigative throughput as caseloads grow.

Recent advances in Large Language Models (LLMs) have demonstrated capabilities in information extraction tasks across law enforcement domains [3, 4]. However, LLMs raise questions regarding their reliability for digital forensic triage. This study evaluated a human‐supervised multi‐model validation approach specifically designed for forensic triage applications.

Current approaches to victim report processing face three fundamental limitations:

First, manual examination of victim reports requires analysts to review each submission individually to identify cryptocurrency wallet addresses, fraudulent platform URLs, and transaction amounts. Processing time increases with caseload, delaying blockchain analysis that depends on timely access to these identifiers.

Second, arbitrary text‐length thresholds fail to distinguish actionable from non‐actionable cases. Brief victim reports may still contain critical wallet addresses enabling immediate blockchain tracing. In our dataset, the shortest report containing a traceable wallet address was only 54 words. This demonstrates that text length alone cannot serve as a reliable proxy for forensic actionability. What matters for forensic triage is not narrative length but the presence of information that enables immediate investigative action.

Third, single‐model LLM approaches lack reliability verification mechanisms. Existing studies demonstrate LLM capabilities in police report processing [3] and forensic documentation [4], but these systems typically rely on single models without cross‐validation. In forensic contexts where extraction errors could misdirect investigations or overlook critical evidence, additional reliability safeguards are essential. Inter‐model consensus serves as a confidence signal, while disagreements trigger human review. This balances extraction efficiency with forensic accountability.

This study contributes a validation‐oriented triage framework for human‐supervised complaint review:

- Information‐Based Forensic Taxonomy: We introduce a classification scheme that prioritizes cases by the presence of traceable forensic identifiers rather than subjective metrics like text length or narrative completeness. Cases containing cryptocurrency wallet addresses (ACTIONABLE_HIGH) receive the highest priority, followed by those with platform URLs and loss amounts (ACTIONABLE_MEDIUM), URLs alone (ACTIONABLE_LOW), and finally those lacking any identifiers (FAIL_NO_IDENTIFIERS).
- Tri‐Model Validation Protocol: We implement cross‐verification across three LLMs where inter‐model consensus serves as a reliability signal for automated processing, while discrepancies flag cases for human review. This differs from traditional ensemble approaches that aggregate outputs; here, disagreement itself constitutes actionable information for forensic quality control.
- Multi‐modal Verification Protocol: We demonstrate vision‐language model (VLM) capabilities in detecting narrative‐evidence discrepancies by comparing victim text statements against screenshot evidence. Cases where textual claims (e.g., “Coinbase platform”) contradict visual evidence (phishing clone websites) are flagged for investigator attention.

This study examines the operational validity of a human‐supervised, multi‐model LLM protocol for forensic triage and lead generation from public complaint narratives. It does not seek to establish preservation, provenance, chain of custody, admissibility, or criminal attribution, but instead focuses on the extraction and prioritization of investigator‐actionable identifiers. We hypothesized that a human‐supervised tri‐model protocol would outperform lexical and pre‐trained NLP baselines on adjudicated triage‐relevant identifier extraction, and that cross‐model disagreement would effectively isolate cases requiring manual review. The study addresses the following research questions:

- RQ1. Can a human‐supervised multi‐model prompt protocol recover adjudicated actionable identifiers from public complaint narratives with higher triage‐relevant accuracy than lexical and pre‐trained NLP baselines?
- RQ2. Can cross‐model disagreement effectively isolate cases requiring prioritized manual verification?
- RQ3. Can auxiliary screenshot analysis surface image‐only identifiers or narrative‐image discrepancies relevant to triage?

## 2 RELATED WORK

### 2.1 Pig butchering fraud and blockchain forensic triage

The term “pig butchering” (杀猪盘, sha zhu pan) originates from Chinese criminal argot, where scammers metaphorically “fatten” victims through trust‐building before financial “slaughter” [1]. Unlike traditional romance scams that request direct monetary transfers, pig butchering operations integrate three distinct phases: (1) initial contact via dating apps with fabricated wealthy personas, (2) gradual relationship cultivation while introducing cryptocurrency “investment opportunities,” and (3) systematic extraction through fake trading platforms that display fabricated profits before blocking withdrawals [5]. Cross [2] documented global expansion of these operations beyond Asia, noting their integration of cryptocurrency platforms creates evidence trails across blockchain systems and web infrastructure requiring coordinated technical analysis.

Recent advances in blockchain forensics have established cryptocurrency wallet addresses as the primary identifier for fund tracing. Park et al. [6] proposed a three‐phase forensic investigation framework emphasizing that wallet addresses enable direct blockchain tracing and actor attribution. Holmes and Buchanan [7] developed a triage‐based framework for Bitcoin wallet forensics, demonstrating that prioritization based on traceability improves investigative efficiency. The Financial Action Task Force [8] guidance confirms that virtual assets may be easier to trace than traditional assets when authorities have appropriate tools.

Rogers et al. [9] introduced the Computer Forensics Field Triage Process Model (CFFTPM), establishing the principle that evidence should be classified by its potential to enable immediate investigative action rather than arbitrary metrics. This triage philosophy informs our taxonomy design, prioritizing cases containing blockchain‐traceable identifiers over those with narrative‐only information.

### 2.2 LLM‐based identifier extraction in forensic contexts

Recent advances demonstrate LLM capabilities in extracting structured information from law enforcement reports. Xing and Chen [3] developed a fine‐tuned LLM system for Chinese police incident reports, achieving F1 scores exceeding 0.85 on entity extraction tasks. However, their approach required substantial labeled training data (thousands of annotated cases), which may be impractical for rapidly emerging fraud types where annotated datasets do not yet exist.

Michelet and Breitinger [4] investigated ChatGPT and Llama for digital forensic report generation, finding that while LLMs can assist with standardized report sections, they exhibit concerning hallucination behaviors when generating technical analysis sections. This underscores the critical need for human verification in forensic LLM applications.

The forensic science community has emphasized validation and error rate quantification for any analytical method introduced into legal proceedings [10]. Traditional approaches rely on inter‐rater agreement between human experts, but recruiting forensic experts to annotate hundreds of victim reports represents significant resource investment, creating tension between validation requirements and automation objectives.

### 2.3 Multi‐model validation and research positioning

Ensemble methods combining multiple models have demonstrated improved robustness across various NLP tasks [11]. However, most ensemble approaches involve model output aggregation rather than discrepancy detection. In forensic contexts, disagreement between models may be more informative than consensus: cases where multiple commercial LLMs produce divergent extractions likely require human expert review regardless of which model is correct. Our tri‐model protocol differs from traditional ensemble learning by flagging disagreements for human attention rather than aggregating outputs, using cross‐model consensus as a confidence indicator for triage priority.

Existing literature demonstrates LLM capabilities in law enforcement text processing [3] and forensic documentation [4], but does not address forensic triage taxonomy design, multi‐model reliability protocols for quality control, multi‐modal consistency verification between narratives and visual evidence, or actionability‐focused evaluation frameworks that distinguish extracting information from extracting actionable intelligence. This study addresses a narrower validation problem: whether human‐supervised multi‐model extraction can recover adjudicated triage‐relevant identifiers from public complaint narratives more consistently than lexical and pre‐trained NLP baselines.

## 3 MATERIALS AND METHODS

Figure 1 shows the extraction method. Victim reports undergo schema‐driven structuring through parallel LLM processing, followed by cross‐model discrepancy identification to support human review prioritization.

**FIGURE 1:**
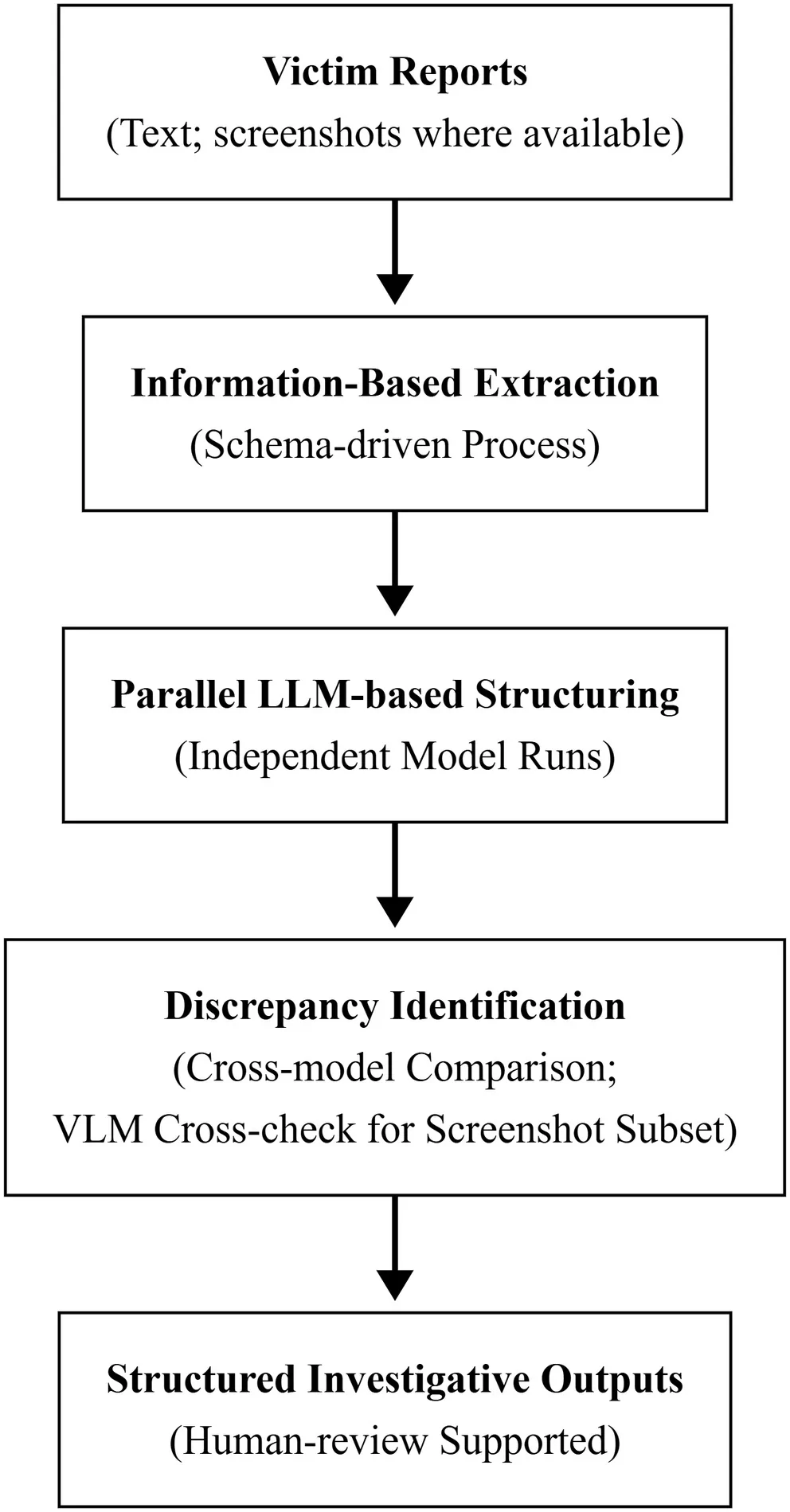
Tri‐model forensic triage. Victim reports (text, with screenshots where available) undergo schema‐driven, information‐based extraction followed by independent, parallel LLM‐based structuring. Screenshot‐containing cases receive an auxiliary vision‐language model (VLM) cross‐check during discrepancy identification, and the resulting structured investigative outputs support human‐review prioritization.

### 3.1 Data acquisition and ethical consideration

This study analyzed 273 pig butchering scam victim reports from the California Department of Financial Protection and Innovation (DFPI) Crypto Scam Tracker, a publicly accessible consumer complaint database. The dataset represents complaints filed between January 2022 and December 2024. Each case contains free‐text victim narratives (50–3000 words) and optional screenshot attachments (25 cases). These anonymized narratives retain the unstructured complexity characteristic of financial fraud reports while mitigating privacy risks. This research was determined exempt from full IRB review as it involves secondary analysis of publicly available, de‐identified data.

### 3.2 Information‐based forensic taxonomy

We defined a rule‐based reference method (Table 1) by extracting explicit and objectively verifiable indicators commonly found in pig butchering scam reports. These indicators require minimal semantic interpretation and can be reliably identified using regular expressions. This method consists of three categories: cryptocurrency wallet identifiers, platform‐related URLs, and monetary loss expressions. The regex module was included as a lexical reference baseline for explicit string recovery, not as a semantic information extraction comparator.

**TABLE 1:** Identifier detection patterns.

Category	Pattern classes	Description/types
Cryptocurrency wallets	14	Blockchain address formats
Platform URLs	3	Full URLs, common domains, suspicious domains
Loss amounts	4	Monetary expressions

The regex approach focuses exclusively on explicit indicators that can be extracted deterministically. It serves as a lexical recovery floor rather than a semantic extraction comparator, establishing the baseline accuracy achievable without contextual interpretation.

Prior research has demonstrated that despite advances in machine learning, rule‐based systems remain prevalent in industry applications requiring interpretability and debuggability [12]. We adopt regex‐based keyword extraction as an operational comparison because most digital forensic analysis tools provide keyword search and indexing as core functionality, and regex‐based pattern matching represents a practical implementation for identifier extraction in operational workflows. Prior work on formal modeling of forensic tool behavior demonstrates that keyword and regex‐based retrieval can be incomplete due to indexing and parsing failures, creating missed evidence risk even when patterns are correctly specified [13]. Furthermore, cybercrime investigations consistently report challenges with high data volumes and insufficient resources for specialized tools and training, underscoring the need for advanced analytical methods [14]. These factors motivate a rigorous comparison against methods that can recover context‐dependent artifacts and quantify disagreement for targeted human review.

The 14 cryptocurrency wallet pattern classes cover major blockchain formats: Ethereum (0x‐prefixed), Bitcoin (Bech32, Legacy, Cash), TRON, Litecoin, XRP, Dogecoin, Solana, Monero, Dash, NEO, and TON. The three URL pattern classes target full URLs with protocol prefixes, common cryptocurrency‐related domain extensions, and suspicious domains mimicking legitimate exchanges. The four loss amount patterns capture dollar‐prefixed expressions, USD‐suffixed amounts, contextual loss descriptions, and shorthand notations (e.g., “$50 k”, “2 million”).

The DFPI Crypto Scam Tracker dataset contains two URL sources: (1) the unstructured “complaint_narrative” field containing victim reports, and (2) a structured “website” field populated by DFPI staff during complaint processing. Notably, 67.8% of cases (*n* = 185) contain website information only in the structured field, not mentioned in the victim narrative. Our identifier extraction combines both sources to maximize forensic intelligence recovery.

The classification is based on the presence of forensic identifiers that enable immediate investigative action (Table 2). Cryptocurrency wallet addresses constitute the highest priority (ACTIONABLE_HIGH) as they enable direct fund‐flow tracing through blockchain explorers [15]. Platform URLs combined with monetary amounts (ACTIONABLE_MEDIUM) support infrastructure analysis and resource prioritization. URLs alone (ACTIONABLE_LOW) provide limited domain intelligence. Cases without identifiers (FAIL_NO_IDENTIFIERS) are non‐actionable. This taxonomy draws on established digital forensic triage principles [9] and cryptocurrency forensics frameworks [6, 7]. The FATF [8] guidance confirms that wallet addresses enable transaction tracing, supporting virtual asset investigations when combined with additional attribution data.

**TABLE 2:** Information‐based classification criteria.

Status	Criteria	Forensic value
ACTIONABLE_HIGH	Wallet address present	Direct blockchain tracing
ACTIONABLE_MEDIUM	URL + monetary amount present	Infrastructure analysis + prioritization
ACTIONABLE_LOW	URL only	Limited domain intelligence
FAIL_NO_IDENTIFIERS	None	Non‐actionable

### 3.3 Extraction schema design

The extraction schema was designed in JSON format to capture forensic identifiers and investigative context from victim narratives, including contextual elements such as initial contact methods, grooming tactics, and investment solicitation patterns. Each LLM output was validated against the predefined schema by an external parsing script; invalid outputs (malformed JSON, missing keys, type violations) triggered automated retries (maximum 3 iterations).

Each prompt included: (1) explicit forensic analyst role definition, (2) extraction targets distinguishing scammer‐controlled artifacts from victim‐owned references, (3) JSON output format with predefined keys, and (4) instructions to return null rather than infer missing information. This design addresses hallucination risks identified in prior forensic LLM applications [4] by constraining outputs to verbatim extraction.

### 3.4 Multi‐model extraction protocol

Table 3 presents the model specifications used in this study.

**TABLE 3:** Model specifications.^a^

Model name	Provider	API identifier	Context window	Max output
Claude Sonnet 4.5	Anthropic	claude‐sonnet‐4‐5	200K tokens	64K tokens
GPT‐5.2	OpenAI	gpt‐5.2	400K tokens	128K tokens
Gemini 3 Pro	Google	gemini‐3‐pro‐preview	1M tokens	64K tokens

*Note*: Controlled variables: Temperature = 0.0, Max Tokens = 4000, Identical system prompts across all models.

^a^ *Source*: Official technical specifications from OpenAI, Anthropic, and Google (As of Jan 2026).

All experiments were conducted via official REST APIs to ensure reproducibility and precise parameter control, avoiding the hidden system prompts and dynamic stochasticity inherent in web‐based chat interfaces. Temperature was set to 0.0 to ensure deterministic outputs, following established best practices for information extraction tasks where reproducibility is critical [16]. The three models from independent providers (Anthropic, OpenAI, Google) were evaluated using identical system prompts to minimize correlated extraction errors and enable fair cross‐model comparison.

### 3.5 Multi‐modal verification: A pilot cross‐check

As an auxiliary cross‐check, we applied a multi‐modal protocol to the 25 cases containing screenshot evidence. We report these results as a pilot finding intended to motivate a dedicated follow‐up study with proper ground truth at scale; these results are not included in the primary F1 evaluation in §4.3, and the pilot was not designed to benchmark multi‐modal extraction performance. For the pilot, we adopted three subroutines:

- Visual Extraction: The model extracted platform names, URLs, and UI elements visible in the screenshots.
- Additional Identifier Discovery: The model identified wallet addresses and platform URLs visible only in screenshot evidence but not mentioned in the complaint narrative text.
- Discrepancy Flagging: Cases where the victim report text claimed a legitimate platform (e.g., “Coinbase”) but the screenshot evidence showed a phishing clone (e.g., “Coinbase‐vip.com”) were flagged as requiring further investigation. This verification protocol flags inconsistencies for investigator review, not automated fraud determination.

Multi‐modal verification employed each model's vision capability to compare narrative content against screenshot evidence. Inconsistent cases were flagged for analyst review to identify potential platform impersonation (e.g., text referencing legitimate services while screenshots display lookalike domains). Primary extraction performance (precision/recall/F1) is evaluated against the adjudicated ground truth derived from complaint narratives and structured fields (*N* = 273). Multi‐modal verification is reported separately on the screenshot subset (*N* = 25) as an auxiliary protocol to identify image‐only identifiers and narrative–evidence discrepancies; these results do not contribute to the primary F1 metrics. Case‐level screenshot materials underlying this cross‐check are provided as Figures S1–S25.

### 3.6 Ground truth annotation

Two annotators independently annotated all 273 cases: one researcher with digital forensics expertise and one active law enforcement investigator with cryptocurrency fraud investigation experience. Three identifier categories were annotated:

- Wallet address (triage‐relevant): Destination address to which the victim reported transferring funds. Victim‐owned addresses and legitimate exchange deposit addresses were excluded from the adjudicated set unless contextual evidence indicated they served as intermediary points in the scam flow.
- Platform URL (triage‐relevant): Domain associated with a suspected fraudulent investment platform as described in the complaint narrative or recorded in the DFPI structured website field. Legitimate exchange domains (e.g., coinbase.com, binance.com) were excluded.
- Reported loss amount (triage‐relevant): Monetary amount the complainant reported as transferred to the scam operation. Fake profits displayed on fraudulent platforms, fee or tax demands not yet paid, and unrealized balances were excluded.

Inter‐annotator agreement was measured using Cohen's *κ*: wallet addresses (*κ* = 0.978), platform URLs (*κ* = 0.988), loss amounts (*κ* = 0.969). Disagreements were resolved by the lead author.

The ground truth dataset contained 43 wallet addresses across 25 cases, 386 platform URLs across 258 cases, and reported losses totaling $53.9 million across 212 cases. Manual annotation required approximately 20 h per annotator for 273 cases, with each extracted identifier cross‐verified against narrative context. We developed a regex module targeting cryptocurrency address patterns, URL patterns, and monetary‐loss expressions, as summarized in Table 1. While it may not represent commercial extraction tools, it demonstrates the limitations of static pattern matching when applied to varied linguistic expressions in fraud narratives. External validation confirmed that all 43 wallet addresses identified through tri‐model consensus (i) conform to the syntactic format of their respective blockchain protocols and (ii) exhibit observable on‐chain transactions via public blockchain explorers. This validation establishes format validity and on‐chain activity, not contextual correctness or legal attribution.

## 4 RESULTS

### 4.1 Pre‐classification distribution

Applying the information‐based forensic taxonomy to the complete DFPI dataset (*N* = 273), we observed the following distribution (Table 4):

**TABLE 4:** Pre‐classification results.

Status	Count	Percentage
ACTIONABLE_HIGH	25	9.2
ACTIONABLE_MEDIUM	201	73.6
ACTIONABLE_LOW	38	13.9
FAIL_NO_IDENTIFIERS	9	3.3
Total	273	100

This distribution reveals that most victim reports (96.7%) contain at least one category of actionable forensic intelligence. However, only 9.2% of cases (25/273) contain the highest‐priority artifact—cryptocurrency wallet addresses—which enable direct blockchain tracing. This finding validates the need for automated triage: manual review of all 273 cases to locate these 25 high‐priority wallets would be inefficient compared to automated pre‐filtering.

The presence of URLs in 264 cases (96.7%) reflects platform information captured from both victim narratives and DFPI‐populated structured fields. However, URL presence alone does not guarantee investigative value: many URLs reference legitimate platforms (e.g., Coinbase.com) used for initial fund transfers before movement to scammer‐controlled addresses, requiring analyst judgment to distinguish platform mentions from actual fraud infrastructure.

### 4.2 Tri‐model identifier extraction performance

Table 5 presents the consolidated extraction results across Claude Sonnet 4.5, GPT‐5.2, and Gemini 3 Pro. All 273 cases were processed; 264 were classified as actionable. Of the 264 cases containing at least one URL, 258 contained URLs meeting the adjudicated fraud‐related platform definition used for ground‐truth annotation.

**TABLE 5:** Tri‐model identifier extraction results.

Model	Cases processed	Unique wallets	Platform URLs	Total loss (USD)
Claude Sonnet 4.5	273	43	508	$53,972,182
GPT‐5.2	273	43	536	$52,964,762
Gemini 3 Pro	273	43	472	$52,242,615

All three models identified 43 unique cryptocurrency wallets. Platform URL extraction counts were Claude 508, GPT 536, and Gemini 472. The difference between highest and lowest was 64 URLs. Model outputs differed in the set of URLs extracted. Total loss amounts were Claude $54 million, GPT $53 million, and Gemini $52 million approximately. The difference between highest and lowest was $1.8 million. All three totals fell between $52.2 M and $54 M for this 273‐case dataset.

Crucially, external blockchain verification confirmed that all 43 wallet addresses identified through tri‐model consensus exhibited valid blockchain format and observable on‐chain activity (100%, 43/43). The complete text extraction pipeline processed all 273 cases, of which 264 were classified as actionable, in approximately 74 min (Claude 38 min, GPT 14 min, Gemini 22 min) with an estimated API cost of approximately $15 USD, demonstrating cost‐effective scalability. Additionally, LLMs extracted contextual fields (contact method, scammer persona, duration) that regex patterns cannot capture. While these fields are captured in the structured JSON output for investigative use, they were excluded from the quantitative analysis as their values require qualitative interpretation.

### 4.3 Performance against ground truth

Table 6 presents extraction performance evaluated against human‐annotated ground truth across all cases.

**TABLE 6:** Performance against adjudicated ground truth (*N* = 273): Regex, spaCy NER, Claude Sonnet 4.5, GPT‐5.2, Gemini 3 Pro.

Identifier	Metric	Regex	spaCy NER	Claude	GPT	Gemini
Crypto Wallet	Precision	0.840	1.000	1.000	1.000	1.000
	Recall	0.977	0.977	1.000	1.000	1.000
	F1‐score [95% CI]	0.903 [0.857,0.946]	0.988 [0.955,1.00]	1.000 [1.00,1.00]	1.000 [1.00,1.00]	1.000 [1.00,1.00]
Platform URL	Precision	0.360	0.790	0.886	0.890	0.893
	Recall	0.584	0.987	0.938	0.933	0.944
	F1‐score [95% CI]	0.446 [0.411,0.480]	0.877 [0.855,0.899]	0.911 [0.886,0.935]	0.911 [0.885,0.935]	0.918 [0.894,0.940]
Loss Amount	Precision	0.367	0.354	0.905	0.886	0.922
	Recall	0.396	0.382	0.849	0.844	0.887
	F1‐score [95% CI]	0.381 [0.317,0.445]	0.367 [0.304,0.431]	0.876 [0.833,0.915]	0.865 [0.819,0.907]	0.904 [0.864,0.939]

*Note*: Regex does not distinguish actual losses from other monetary amounts. 95% confidence intervals computed by case‐level bootstrap resampling (*B* = 10,000, seed = 42). The 1.000 wallet F1‐scores should be interpreted in light of the modest positive sample (43 addresses across 25 cases) and the structurally distinctive surface form of blockchain addresses, which reduces ambiguity for both rule‐based and LLM extractors.

Wallet address extraction achieved F1 = 1.000 for all three models, compared to F1 = 0.903 for regex. Because the adjudicated wallet label was limited to destination addresses identified in context, regex and spaCy results should be interpreted as lexical or entity‐level recovery under a context‐dependent target definition rather than as direct contextual competitors. All three LLMs achieved perfect recall against this adjudicated standard.

URL extraction achieved F1 = 0.911–0.918 across models. Variation reflected differences in extracting and normalizing fraud‐related platform references under the adjudicated URL definition. spaCy NER achieved the highest recall (0.987) but lower precision (0.790) due to inclusion of legitimate service domains, illustrating the triage‐relevant filtering that contextual models provide.

Loss amount extraction achieved F1 = 0.865–0.904 across models. Both regex and spaCy NER extracted monetary expressions indiscriminately (F1 ≤0.381), capturing fee demands, displayed profits, and partial deposits alongside reported losses. LLM‐based extraction achieved higher F1 by applying contextual filtering consistent with the adjudicated loss definition, as illustrated in Case #58 below.

### 4.4 Multi‐modal verification results

Table 7 presents additional identifier discovery results for the 25 cases containing screenshot evidence. Representative screenshots illustrating image‐only identifier recovery and narrative–screenshot discrepancies are provided in Figures S1–S25.

**TABLE 7:** Auxiliary screenshot cross‐check results (*N* = 25).

Discovery type	Claude sonnet 4.5	GPT‐5.2	Gemini 3 pro
Image‐only wallet addresses	4 (16%)	3 (12%)	3 (12%)
Image‐only platform URLs	13 (52%)	11 (44%)	13 (52%)

Within this pilot subset, multi‐modal analysis surfaced identifiers not present in complaint narratives. These pilot observations suggest that screenshot analysis may supplement text‐based extraction; the operational value, error rates, and ground‐truth construction at scale warrant a follow‐up study designed specifically for this purpose. For example, case #24 showed an inconsistent result, with text referencing “Riot Blockchain” while the screenshot displayed “lockchains.com”—a domain impersonation pattern flagged for investigation.

### 4.5 Investigator deliverables data

Table 8 presents an example of the investigator deliverables generated by the framework.

**TABLE 8:** Investigator deliverables.

File	Records	Purpose
case_priority_report.csv	273	Triage all cases by priority
wallet_trace_list.csv	43	Blockchain‐ready wallet list
flagged_cases.csv	82 (31%)	Cases requiring human review

The validation approach generated three standardized outputs for investigators: (1) Case Priority Report (273 records) provides triage classification for all cases (2) Wallet Trace List (43 addresses) enables direct blockchain analysis (3) Flagged Cases (82 cases, 31%) identify cases where models disagreed on URL extraction or loss amounts, prioritizing them for human verification. Wallet addresses, which achieved unanimous consensus across all models, did not trigger flagging.

To illustrate the extraction pipeline, we present Case #58 as Table 9. The complaint narrative mentioned three monetary amounts: $29,000 (commission demand), $48,000 (tax demand), and $4200 (reported loss). Regex selected the first dollar amount ($29,000), while all three LLMs correctly identified $4200 as the actual loss through semantic context. Beyond the text‐based ground truth, the auxiliary multi‐modal cross‐check on this case also surfaced a Bitcoin wallet (bc1qwrxt8…76fw0) visible only in the attached screenshot—a blockchain trace absent from the complaint narrative. As noted in §4.4, image‐only discoveries are reported separately from the primary F1 metrics and are not aggregated with the text‐based ground truth.

**TABLE 9:** Illustrative example of context‐dependent loss adjudication and screenshot‐only identifier discovery (case #58).

Identifier	Ground truth (text)	Ground truth (image)	Regex	Claude sonnet 4.5	GPT‐5.2	Gemini 3 pro
Wallet address	0	1	0 (text only)	1 (image)	1 (image)	1 (image)
Platform URLs	1	n/a	1	2	2	1
Loss amount	$4200	n/a	$29,000	$4200	$4200	$4200

*Note*: ‘Ground Truth (Text)’: adjudicated ground truth derived from the complaint narrative used for the primary F1 evaluation. ‘Ground Truth (Image)’: ground truth derived from screenshot analysis (auxiliary multi‐modal cross‐check, §4.4); image‐only discoveries are not included in the primary F1 benchmark. The wallet bc1qwrxt8…76fw0 was visible only in the attached screenshot.

Case #58 illustrates two complementary capabilities: (1) semantic disambiguation of multiple monetary amounts against the text‐based adjudicated ground truth (Table 6 primary F1), and (2) auxiliary multi‐modal recovery of identifiers visible only in screenshots (§3.5 protocol; §4.4 results), reported separately from primary F1.

## 5 DISCUSSION

### 5.1 Forensic triage: Actionability over coverage

The information‐based taxonomy classified 96.7% of cases (264/273) as actionable based on forensic identifier presence. This distribution contrasts with text‐length filtering approaches that would retain verbose narratives lacking traceable artifacts while excluding brief reports containing wallet addresses.

The 25 ACTIONABLE_HIGH cases (9.2%) represent the subset enabling direct blockchain forensic analysis. Automated pre‐classification concentrated investigative resources on these high‐priority cases without requiring manual review of all 273 narratives. Most cases (201, 73.6%) were classified as ACTIONABLE_MEDIUM, containing platform URLs and loss amounts that support infrastructure analysis and cross‐case pattern identification. ACTIONABLE_LOW cases (38, 13.9%) provide domain intelligence for threat feeds and platform blocklists, even without documented financial losses. Nine cases (3.3%) were classified as FAIL_NO_IDENTIFIERS because they provided no traceable technical identifiers. This approach prioritizes forensic traceability over information completeness. Cases lacking wallet addresses, URLs, or loss amounts may retain value for victimology research but offer no immediate blockchain tracing capability. Notably, FAIL_NO_IDENTIFIERS cases may still contain platform names, scammer personas, or contact methods that LLM extraction could recover. The current taxonomy prioritizes technical traceability over information completeness. Future extensions could process these cases for contextual intelligence.

### 5.2 Interpreting the gap between pre‐trained NLP and LLM‐based extraction

The most pronounced performance gap between off‐the‐shelf NLP and LLM‐based extraction appeared in loss amount identification (spaCy F1 = 0.367 vs. LLM F1 = 0.865–0.904). Pre‐trained NER models extracted monetary expressions without distinguishing reported losses from fee demands, displayed profits, or partial deposits. For URLs, spaCy achieved higher recall (0.987) than LLMs (0.933–0.944) but lower precision (0.790 vs. 0.886–0.893), capturing legitimate service domains alongside fraud‐related platforms. These patterns indicate that string‐level recovery is necessary but insufficient for triage‐relevant extraction, where contextual filtering determines operational utility.

### 5.3 Cross‐model consensus analysis

The tri‐model protocol exhibits an artifact‐specific reliability profile that, in our reading, is the practically informative finding from the cross‐model comparison. For wallet addresses, the three models converged on identical extractions (F1 = 1.000), and all 43 consensus addresses cleared format validity and on‐chain activity checks on their respective blockchains. We interpret this as a high‐confidence triage signal: the consensus wallet output can be used to prioritize analyst review and initial blockchain‐tracing preparation, but it does not remove the need for human verification before investigative or evidentiary use, and downstream attribution or chain‐of‐custody assessment remains outside the extraction benchmark. The principal value of the tri‐model design therefore lies less in raw score maximization than in providing artifact‐stratified confidence signals that direct manual review toward cases the framework cannot resolve without human verification.

URL extraction achieved F1 = 0.911–0.918 across models. Variation occurred where models applied different thresholds for fraudulent platform identification—partial domains, obfuscated URLs, and ambiguous platform references produced inconsistent extraction boundaries. Loss amount extraction achieved F1 = 0.865–0.904, with total estimates showing 3% variance across models, consistent with different interpretations of multi‐deposit scenarios.

The divergent patterns between identifier types inform operational deployment. Wallet addresses, exhibiting high inter‐model agreement and confirmed blockchain format validity, represent high‐confidence triage outputs suitable for prioritized blockchain analysis under analyst review. URLs, showing greater extraction variance, require human review before infrastructure investigation. This artifact‐specific reliability profile allows investigators to calibrate trust levels accordingly. However, consensus indicates inter‐model agreement rather than correctness—models trained on similar corpora may exhibit correlated errors. The protocol therefore treats consensus as a confidence signal that reduces, but does not eliminate, human oversight requirements.

While all models achieved identical wallet extraction, URL and loss extractions varied across models. The review queue analyzed in this study was generated by the pipeline's case‐level issue flag. A case was routed to human review when the pipeline recorded a cross‐model discrepancy in fraud‐related URL outputs or reported‐loss outputs. For URLs, this included differences in normalized URL values or in the number of fraud‐related URL candidates extracted across models. For reported losses, this included different normalized USD values or null/non‐null conflicts across models. Wallet addresses, which achieved unanimous consensus in Table 6, produced no flags. This operational rule produced 82 flagged cases: 74 URL‐only, 5 loss‐only, and 3 involving both URL and loss discrepancies (31% of the 264 actionable cases). The flagging rule was applied to the existing model outputs; flagged‐case correctness in Table 10 was then evaluated against the adjudicated ground truth. This post hoc analysis does not change the primary F1 benchmark reported in §4.3.

**TABLE 10:** Per‐model accuracy on disagreement‐flagged cases (*N* = 82).

Disagreement type	*N*	Claude correct	GPT correct	Gemini correct	At‐least one correct
URL only	74	25 (33.8%)	27 (36.5%)	25 (33.8%)	29 (39.2%)
Loss only	5	3 (60.0%)	2 (40.0%)	2 (40.0%)	5 (100%)
Both	3	1 (33.3%)	0 (0.0%)	0 (0.0%)	1 (33.3%)
Total flagged	82	29 (35.4%)	29 (35.4%)	27 (32.9%)	35 (42.7%)

*Note*: “Correct”: the model's extracted set or value matches the adjudicated ground truth at the case level. Cases where no model satisfied the criterion (47 of 82, 57.3%) constitute the residual queue. These figures were computed by post hoc re‐aggregation of existing model outputs against the same adjudicated ground truth used for Table 6.

To characterize this operational flag analytically, we re‐aggregated the same outputs under a formalized signal: a URL‐disagreement on a case corresponds to a non‐empty symmetric set difference between any two models' normalized URL sets (lowercased, scheme‐ and trailing‐slash‐stripped, deduplicated), and a loss‐disagreement corresponds to a pre‐specified operational tolerance—maximum pairwise difference exceeding 5% of the largest reported value—to accommodate rounding and partial‐deposit aggregation variance. Table 10 reports per‐model correctness for these 82 flagged cases against the same adjudicated ground truth used for Table 6.

The flagged cases cluster into three error families. (a) Boundary disambiguation: subdomain vs. root‐domain choice, trailing‐path retention, and IDN normalization differences. (b) Obfuscated or paraphrased references: typosquatted lookalikes, partial URLs in narrative, and domain mentions split across sentences. (c) Multi‐deposit aggregation: layered transfers reported across multiple time points, where model‐specific aggregation rules diverge. Three patterns are visible in Table 10. First, when models disagree only on loss, at least one model recovers the adjudicated amount in this small subset (*n* = 5). Second, when models disagree only on URLs (*n* = 74), at‐least‐one accuracy falls to 39.2%—the dominant residual queue. Third, the three combined‐flag cases (*n* = 3; interpretation limited by small sample) illustrate a conservative limit on automated triage: only one model (Claude) matched the full ground truth in this subset. The disagreement queue therefore serves as a quality‐control mechanism—directing analyst attention toward systematically ambiguous extractions—rather than as a residual error signal alone.

Each identifier type carries distinct error consequences for investigative workflows. Wallet address false positives may misdirect blockchain tracing toward uninvolved parties. URL overinclusion risks contaminating infrastructure investigations with legitimate service providers. Loss amount misclassification can distort triage priority rankings, potentially delaying high‐value cases. The disagreement queue functions as a quality‐control mechanism: cases flagged by inter‐model divergence receive mandatory human review before downstream action, reducing the propagation of extraction errors into investigative decisions.

### 5.4 Practical implementation

The validation approach produces standardized CSV outputs compatible with existing forensic workflows, addressing a critical gap in LLM‐based tools requiring manual interpretation. Outputs integrate directly with blockchain analysis platforms via wallet address exports. The 31% flagging rate (82/264 cases) represents model disagreement triggering human review, adjustable based on organizational risk tolerance. To contextualize operational impact, we report a pipeline‐stage time comparison. Adjudicated annotation of all 273 cases for identifier extraction—wallet addresses, platform URLs, and loss amounts—required approximately 20 h per annotator (conservative, given non‐native English annotation). The automated tri‐model pipeline completed pipeline‐stage extraction in 74 min (Claude: 38 min; GPT‐5.2: 14 min; Gemini 3 Pro: 22 min) at an API cost of approximately $15 USD, corresponding to a 94% reduction in pipeline‐stage processing time, not in total end‐to‐end workflow time. The 82 flagged cases (31%) explicitly remain in the human‐review queue: at the practitioner‐time estimate of approximately 2 h per case stated in the Introduction, this queue adds on the order of 164 h of mandatory investigator review before downstream action. The efficiency figure therefore refers to the automated extraction component, separate from—and complementary to—the human verification stage that the framework requires by design.

We intentionally evaluate zero‐shot, prompt‐driven extraction using commercial LLM APIs rather than fine‐tuning domain‐specific models because pig butchering scam narratives exhibit rapid linguistic drift and heterogeneous reporting formats, while labeled forensic training data remain scarce and sensitive. Comparative evidence from adjacent empirical settings indicates that fine‐tuning can be infeasible in cold‐start conditions due to dataset requirements and cost, whereas prompting strategies are a practical alternative when task‐specific supervision is limited [17]. Moreover, fine‐tuning on sensitive corpora can amplify privacy and memorization risks, which is particularly problematic for victim reports containing personal information [18].

### 5.5 Operational security considerations

The commercial API‐based architecture evaluated in this study raises operational security concerns for law enforcement deployment. When victim statements are transmitted to external servers operated by commercial API providers, several data sovereignty issues emerge that investigators must address before operational adoption.

First, unpublished investigative information may be exposed to third‐party infrastructure. Victim narratives in active investigations often contain wallet addresses, platform URLs, and transaction details that have not been publicly disclosed. Transmitting such data to commercial APIs potentially compromises investigative confidentiality, particularly in cases involving ongoing surveillance or coordinated international operations.

Second, data retention policies vary across API providers. While major providers offer data processing agreements that prohibit training on customer inputs, the specific retention periods, access controls, and jurisdictional storage locations may conflict with agency‐specific information security requirements. Organizations subject to criminal justice information services (CJIS) compliance or equivalent national standards must verify that API usage aligns with existing data handling protocols.

Three mitigation strategies warrant consideration for operational deployment:

- Local LLM deployment: Open‐source models can be deployed on‐premises, eliminating external data transmission entirely. Recent work has demonstrated the feasibility of domain‐specific local LLMs for digital forensics applications [19], though such approaches require computational infrastructure and may exhibit different performance characteristics than frontier commercial models. Comparative evaluation of fine‐tuned local models versus commercial API‐based extraction represents a priority for future research, requiring dedicated model‐selection rationale, quantization and hardware specification, prompt and JSON‐schema enforcement equivalent to commercial APIs, and a paired evaluation protocol against the same adjudicated ground truth used here—a scope appropriate to a stand‐alone study rather than to a single supplementary comparator.
- Pre‐transmission anonymization: Preprocessing pipelines could mask sensitive identifiers (names, addresses, phone numbers) before API transmission, with automated restoration in extracted outputs. This approach preserves commercial model capabilities while reducing exposure of personally identifiable information. However, wallet addresses and platform URLs—the primary extraction targets—cannot be masked without defeating the extraction purpose.
- Enterprise API agreements: Commercial providers offer enterprise contracts with explicit data retention prohibitions, dedicated infrastructure, and compliance certifications. These agreements may satisfy institutional security requirements while maintaining access to commercial model capabilities with additional costs.

The present study utilized publicly available, regulator‐anonymized DFPI data, so these concerns are mitigated in our experimental protocol. They remain critical considerations for operational deployment on active cases, and agencies considering such deployment must conduct jurisdiction‐specific security assessments before processing active case materials through external APIs. Wallet‐address extraction produces potential leads for subsequent blockchain forensic tracing; the present study did not perform transaction‐level blockchain analysis, attribution, or evidentiary linking.

### 5.6 Limitations and future work

Several limitations should be noted. First, the dataset comprises complaints submitted to a single regulatory agency (California DFPI, 2022–2024), which may limit generalizability across jurisdictions and temporal contexts. Classification accuracy depends on the completeness of victim‐reported information; cases lacking explicit identifiers were excluded regardless of underlying fraud severity.

Second, the experimental protocol employed single‐iteration extractions at deterministic temperature settings without repeated sampling to quantify variance. Bootstrap 95% confidence intervals (B = 10,000) were computed to quantify measurement uncertainty, though these reflect resampling variance rather than repeated experimental runs. Human annotation covered all 273 cases with two independent annotators. Inter‐rater reliability was measured using Cohen's *κ* prior to disagreement adjudication.

Third, the linguistic and regulatory context of this study may limit cross‐jurisdictional applicability. The DFPI dataset comprises English‐language complaints submitted under California's consumer protection framework. Pig butchering scams operate globally, with victim reports filed in diverse languages (Korean, Japanese, German) and regulatory environments with varying disclosure requirements. Narrative structures, monetary expression formats, and platform naming conventions may differ substantially across jurisdictions. Future validation should examine multi‐lingual datasets to assess whether zero‐shot extraction performance generalizes beyond English‐language reports.

Fourth, commercial API‐based models undergo periodic updates that may alter extraction behavior over time, potentially affecting long‐term reproducibility of reported performance metrics. Future work should evaluate open‐source alternatives to establish version‐stable benchmarks independent of proprietary model evolution. Periodic re‐evaluation at 6–12 month intervals would document how successive model improvements affect operational extraction performance.

Fifth, baseline comparators have inherent scope limitations. Regex serves as a lexical reference baseline demonstrating pattern‐matching boundaries, not as a strong semantic information extraction comparator. Similarly, spaCy NER is a pre‐trained general‐purpose model, not a domain‐specific fine‐tuned pipeline optimized for fraud complaint processing. No OCR comparator was included for multi‐modal screenshot analysis; the 25 screenshot cases represent an auxiliary consistency check rather than a systematic evaluation of image‐based extraction. Blockchain validation confirms address format and on‐chain transaction activity but does not establish contextual correctness, legal attribution, or evidentiary chain of custody. This study makes no claim regarding admissibility, provenance, or attribution—all outputs require human verification before investigative or legal action. Finally, no active‐case deployment evaluation was conducted; reported metrics reflect retrospective analysis of publicly adjudicated complaints.

Sixth, the present validation is scoped to commercial API‐based extraction. A direct comparison against open‐source local models would extend the operational picture, but a methodologically defensible comparison requires nontrivial choices in model selection, quantization, hardware budget, prompt and schema enforcement under the deterministic temperature setting used here, and a paired evaluation protocol against the same adjudicated ground truth and case‐level disagreement rule. We deliberately exclude such a comparator from this validation to avoid an under‐specified single‐model evaluation that could mischaracterize either commercial or local systems, and we identify a paired local‐versus‐commercial validation as a stand‐alone follow‐up study, aligned with the multi‐modal cross‐check discussion in §4.4.

Future research should examine cross‐jurisdictional datasets to establish external validity, conduct repeated sampling protocols to quantify measurement uncertainty, and integrate automated blockchain verification mechanisms. Temporal stability analysis is warranted as fraud methodologies evolve. Beyond fraud investigations, this approach may extend to mass‐incident scenarios requiring rapid damage assessment across large victim populations, such as court‐ordered individual loss calculations in cryptocurrency exchange collapses. Additionally, the extraction schema captures contextual fields (scammer personas, contact methods, grooming tactics) excluded from this analysis; future work could leverage these for modus operandi classification and criminal intelligence profiling.

## 6 CONCLUSION

This study evaluated a multi‐model LLM validation approach to extract forensic identifiers from 273 pig butchering scam victim reports. For the ground truth, human annotation of all 273 cases achieved inter‐rater reliability of *κ* = 0.969–0.988. The information‐based taxonomy classified 96.7% of cases as actionable based on wallet addresses, platform URLs, or loss amounts, with 9.2% containing cryptocurrency wallets enabling blockchain tracing. All three models (Claude Sonnet 4.5, GPT‐5.2, Gemini 3 Pro) identified 43 unique wallet addresses. Platform URL extraction ranged from 472 to 536 URLs across models. Loss amount aggregations ranged from $52.2 M to $54 M. Evaluation against ground truth achieved F1 = 1.000 for wallet addresses, F1 = 0.911–0.918 for URLs, and F1 = 0.865–0.904 for loss amounts. An auxiliary multi‐modal cross‐check on 25 screenshot‐containing cases is reported as a pilot observation rather than a primary result; image‐only identifiers (wallet addresses 12–16%; platform URLs 44–52%) point to a future study with proper ground truth at scale. This validation approach positions LLMs as triage tools requiring human oversight. The core contribution is not extraction alone but measurable reliability: ground truth validation quantifies extraction accuracy, while cross‐model consensus and external blockchain verification provide confidence signals for analyst review prioritization. Although this study demonstrates batch processing efficacy for retrospective analysis (273 cases in 74 min at approximately $15 USD), the reported reduction applies to the automated pipeline component, not to the full end‐to‐end workflow, which by design routes flagged cases (82/264, 31%) to mandatory human review. At the practitioner‐time estimate stated in the Introduction, this flagged queue adds on the order of approximately 164 h of mandatory investigator review before downstream action. Per‐case extraction averaged approximately 16 s; future work should evaluate real‐time single‐case processing for intake workflows and validate this approach across jurisdictions. The tri‐model protocol presented here offers a practical path toward balancing extraction efficiency with forensic accountability. The present results support the practical feasibility of human‐supervised intake‐stage triage on a public complaint corpus. The framework should be interpreted as triage support and lead generation under human oversight, not as a substitute for forensic examination, evidentiary decision‐making, or attribution.

## FUNDING INFORMATION

This work was supported by the Korea Institute of Police Technology (KIPoT), funded by the Korean National Police Agency (KNPA), grant RS‐2026‐25535833, for Development of an Integrated Narcotics Investigation System Linked to the Dark Web and Virtual Asset Transaction Tracing.

## CONFLICT OF INTEREST STATEMENT

The authors declare no conflict of interest.

## ETHICS STATEMENT

This study used publicly available, fully redacted complaint records published by the California Department of Financial Protection and Innovation (DFPI). No identifiable personal information was processed. IRB review was not required.

## AI DECLARATION

Claude Sonnet 4.5 (Anthropic), GPT‐5.2 (OpenAI), and Gemini 3 Pro (Google) were used as experimental subjects in the data extraction pipeline evaluated in this study. Language editing assistance was provided by AI tools; the authors reviewed and revised all content and take full responsibility for the published article.

## Supporting information


**Figure S1.** Screenshot evidence for Case #2.
**Figure S2.** Screenshot evidence for Case #9.
**Figure S3.** Screenshot evidence for Case #27.
**Figure S4.** Screenshot evidence for Case #34.
**Figure S5.** Screenshot evidence for Case #43.
**Figure S6.** Screenshot evidence for Case #47.
**Figure S7.** Screenshot evidence for Case #48.
**Figure S8.** Screenshot evidence for Case #66.
**Figure S9.** Screenshot evidence for Case #67.
**Figure S10.** Screenshot evidence for Case #68.
**Figure S11.** Screenshot evidence for Case #69.
**Figure S12.** Screenshot evidence for Case #91.
**Figure S13.** Screenshot evidence for Case #107.
**Figure S14.** Screenshot evidence for Case #111.
**Figure S15.** Screenshot evidence for Case #120.
**Figure S16.** Screenshot evidence for Case #135.
**Figure S17.** Screenshot evidence for Case #136.
**Figure S18.** Screenshot evidence for Case #137.
**Figure S19.** Screenshot evidence for Case #139.
**Figure S20.** Screenshot evidence for Case #158.
**Figure S21.** Screenshot evidence for Case #159.
**Figure S22.** Screenshot evidence for Case #160.
**Figure S23.** Screenshot evidence for Case #161.
**Figure S24.** Screenshot evidence for Case #162.
**Figure S25.** Screenshot evidence for Case #163.

## Data Availability

The dataset is publicly available from the California DFPI Crypto Scam Tracker (https://dfpi.ca.gov/crypto‐scam‐tracker). The demo application, prompt templates, and extraction schema used in this study are archived at Zenodo: https://doi.org/10.5281/zenodo.21282425.
