# Supplementary material for: Human‐supervised LLM triage of pig butchering complaints: A validation study of multi‐model identifier extraction
Source: J Forensic Sci. 2026 Jul 22;71(6):2747–59. doi: 10.1111/1556-4029.70411 (PMC13736209; doi:10.1111/1556-4029.70411)
Supplement: Supplementary file 1 [file JFO-71-2747-s001.pdf]

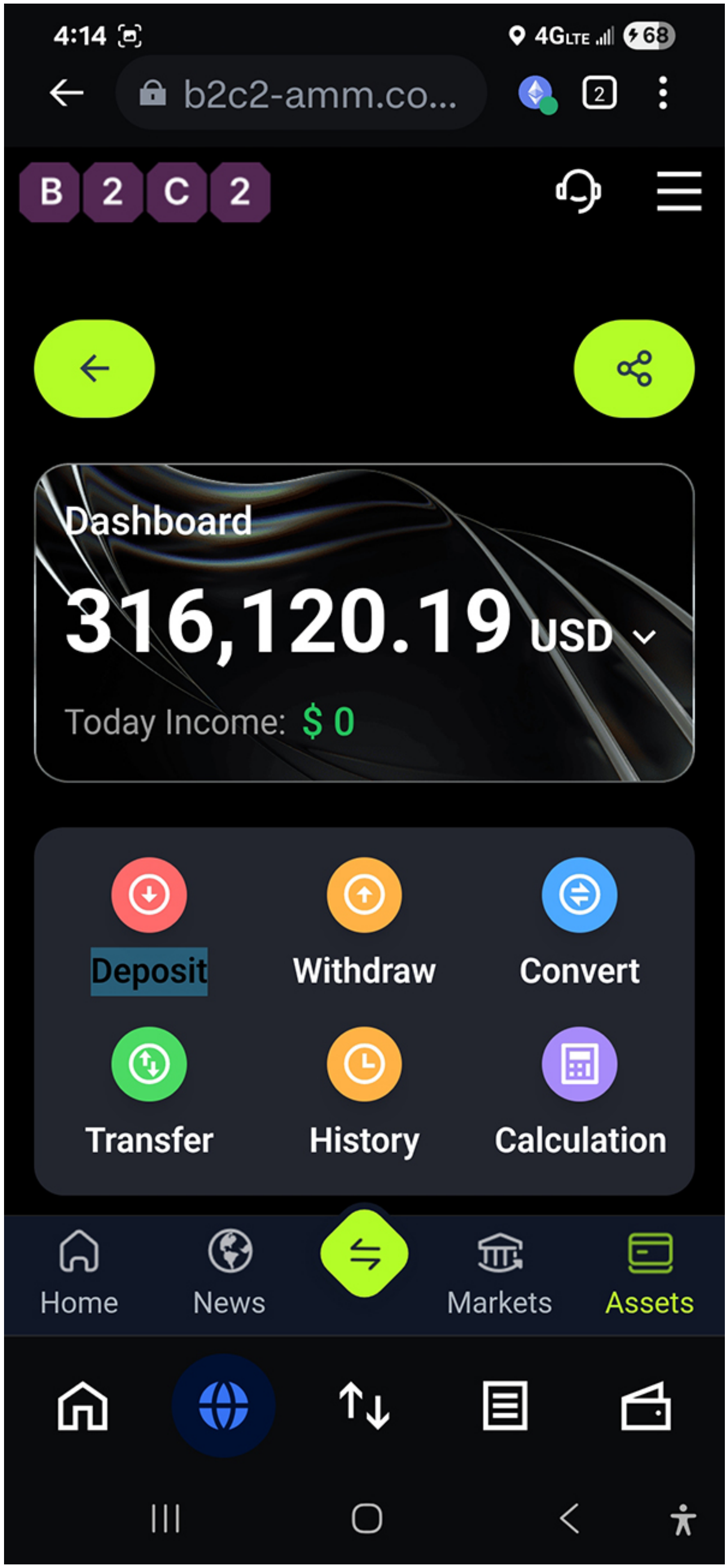

FIGURE S1. Screenshot evidence for Case #2.

Source: DFPI Crypto Scam Tracker (public). File: pb_001_case_002.jpg

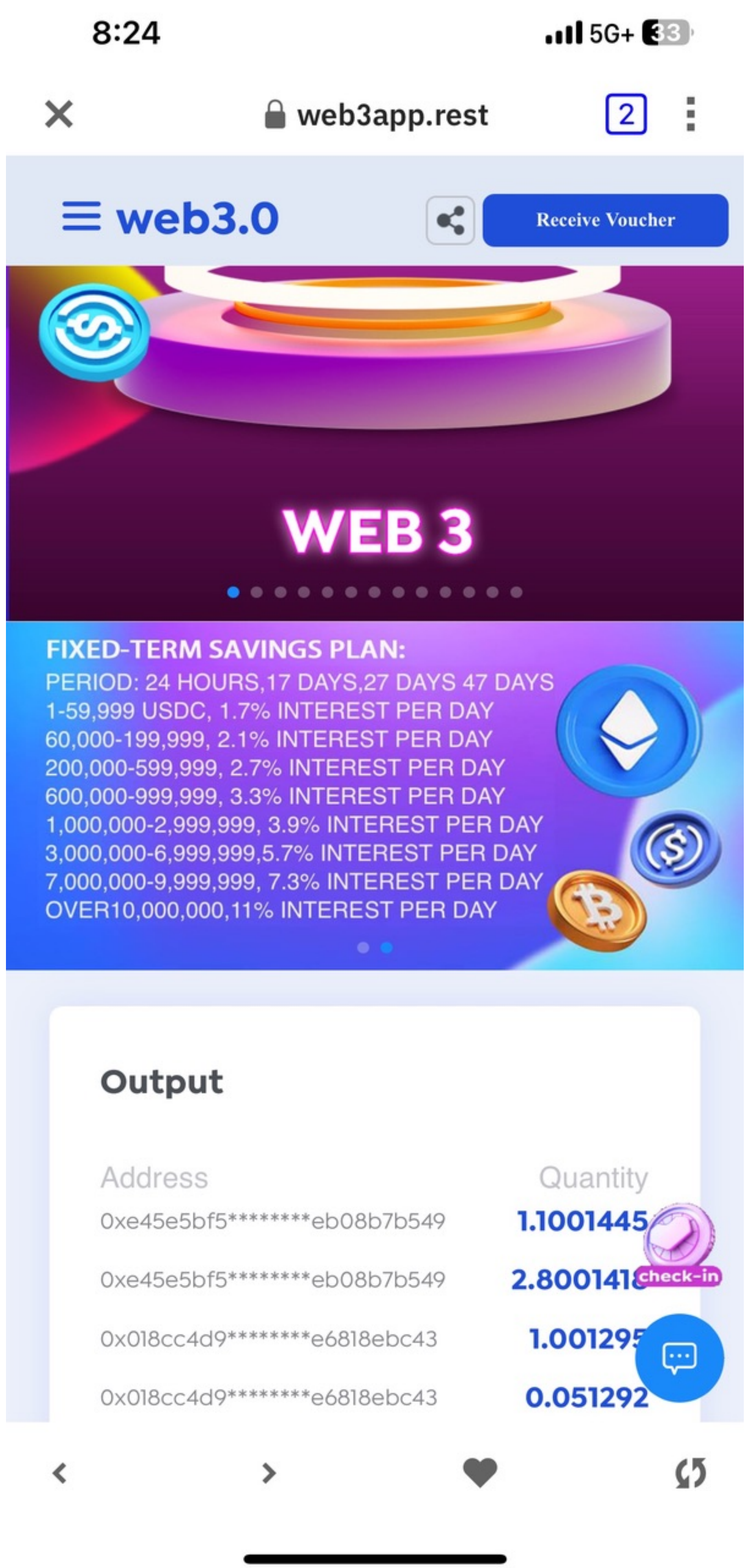

FIGURE S2. Screenshot evidence for Case #9.

Source: DFPI Crypto Scam Tracker (public). File: pb_002_case_009.jpeg

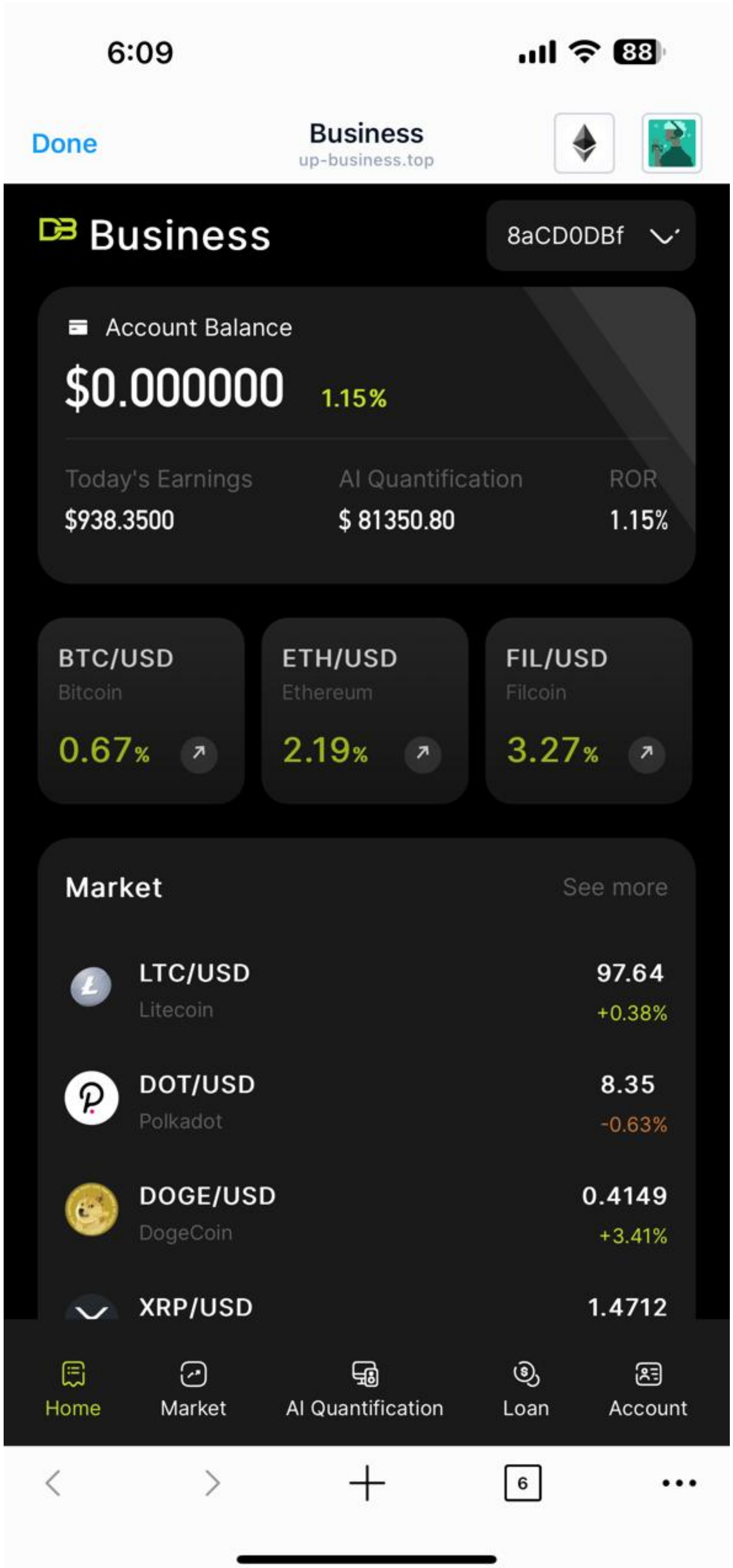

FIGURE S3. Screenshot evidence for Case #27.

Source: DFPI Crypto Scam Tracker (public). File: pb_008_case_027.jpeg

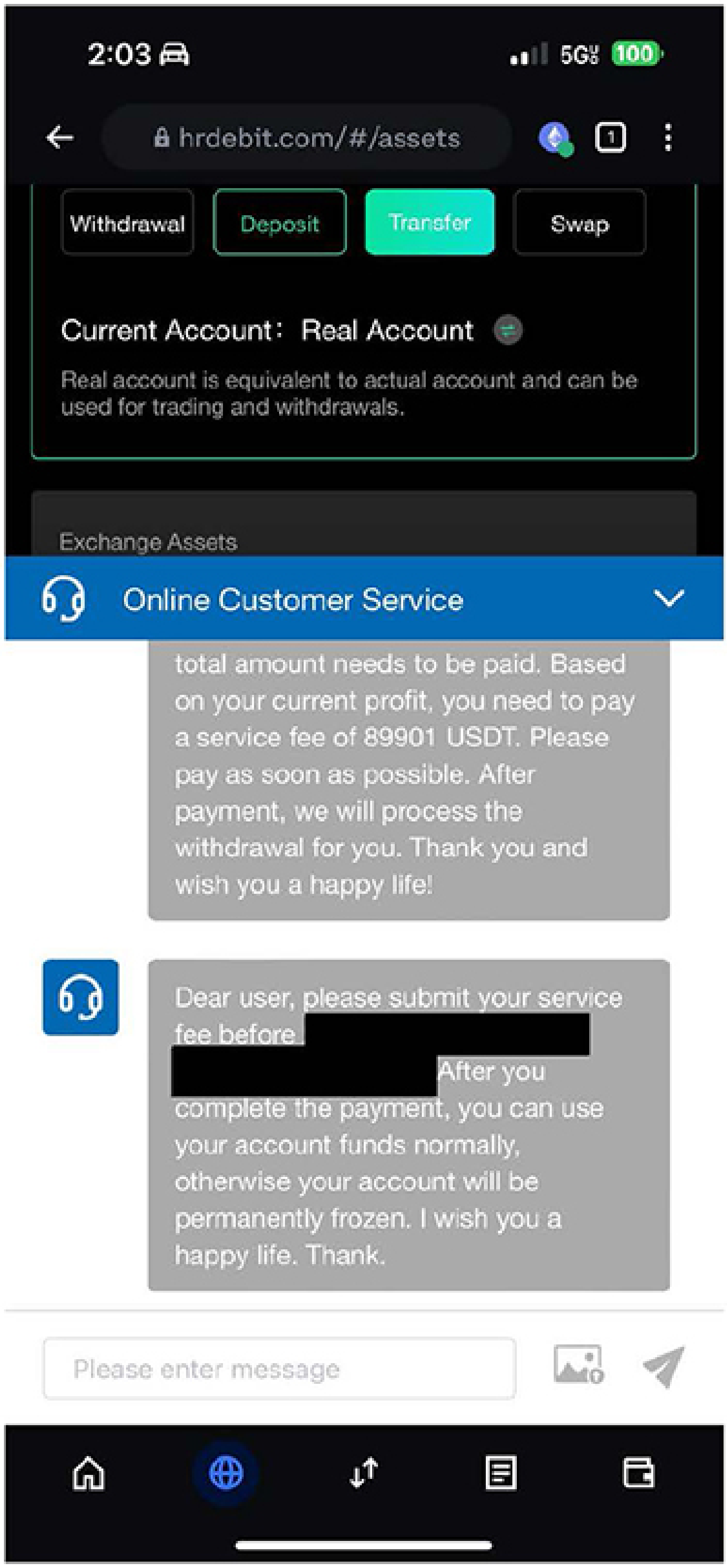

FIGURE S4. Screenshot evidence for Case #34.

Source: DFPI Crypto Scam Tracker (public). File: pb_009_case_034.jpg

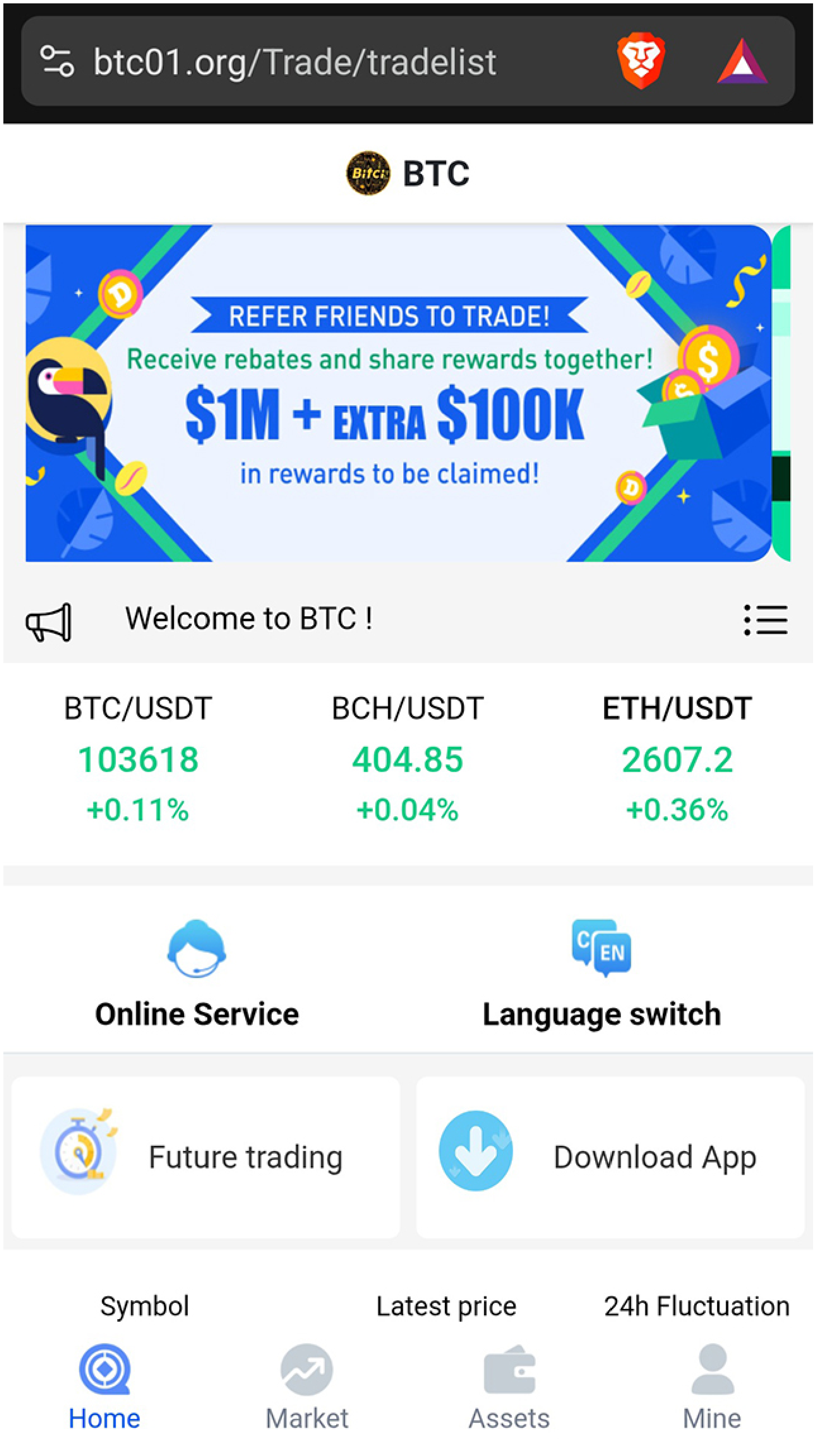

FIGURE S5. Screenshot evidence for Case #43.

Source: DFPI Crypto Scam Tracker (public). File: pb_014_case_043.jpg

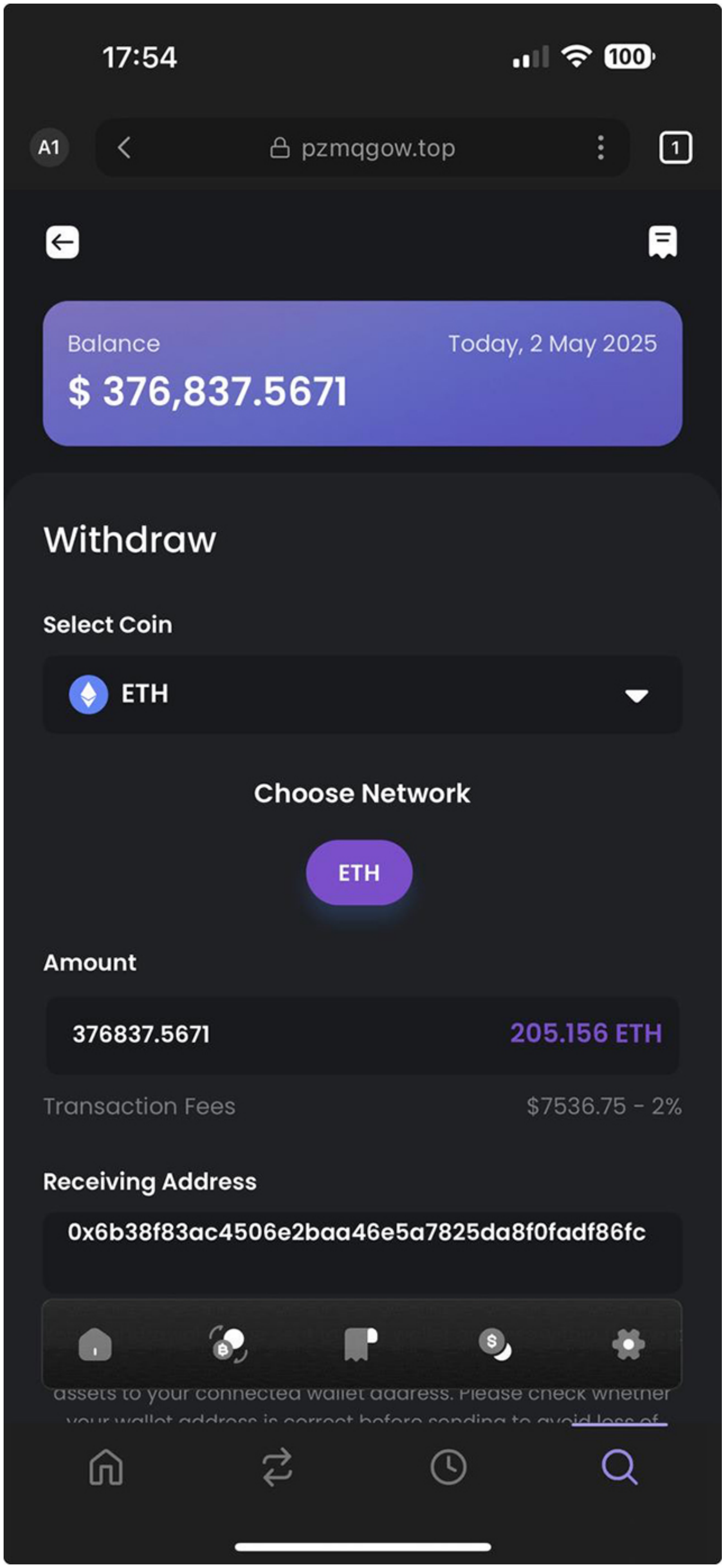

FIGURE S6. Screenshot evidence for Case #47.

Source: DFPI Crypto Scam Tracker (public). File: pb_015_case_047.jpeg

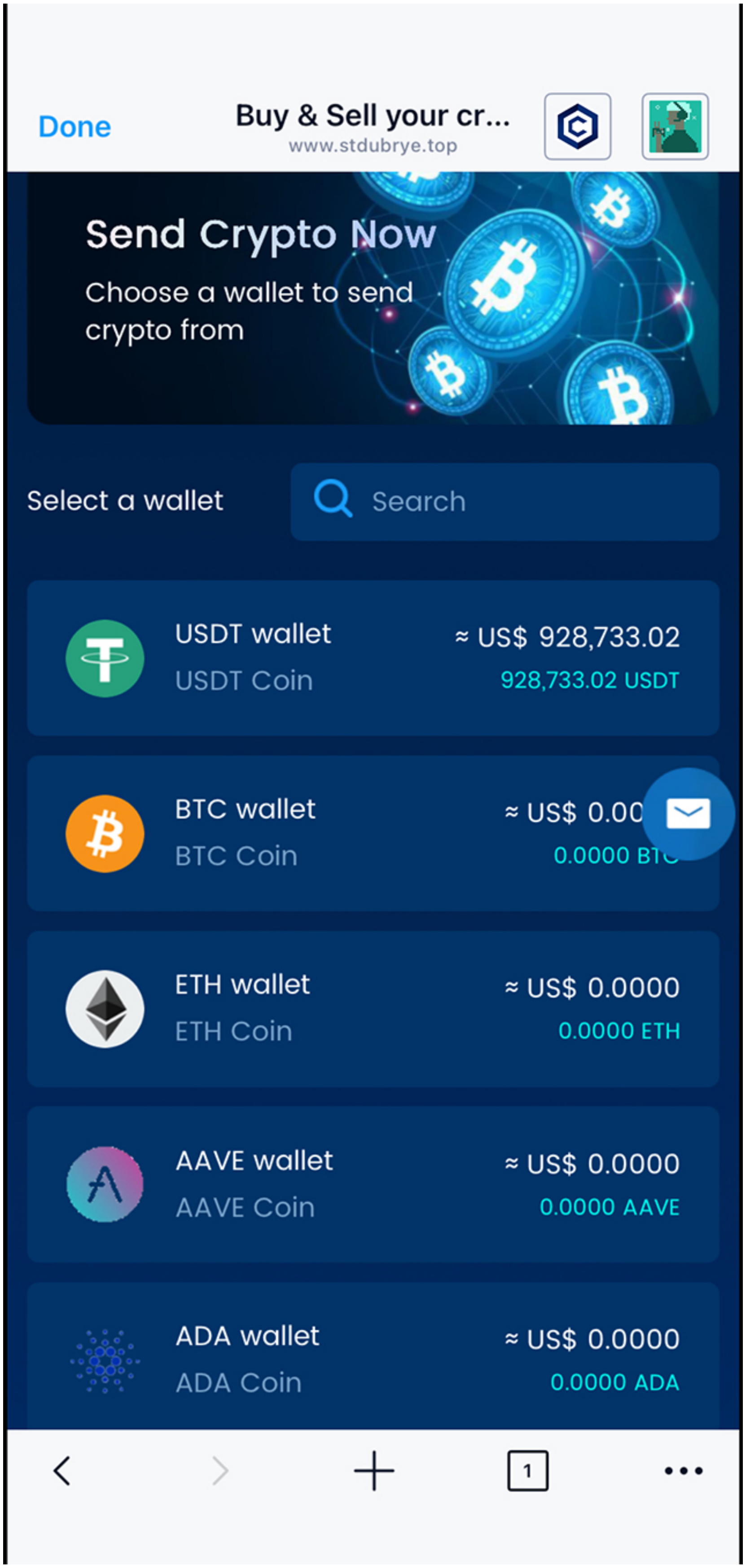

FIGURE S7. Screenshot evidence for Case #48.

Source: DFPI Crypto Scam Tracker (public). File: pb_016_case_048.jpeg

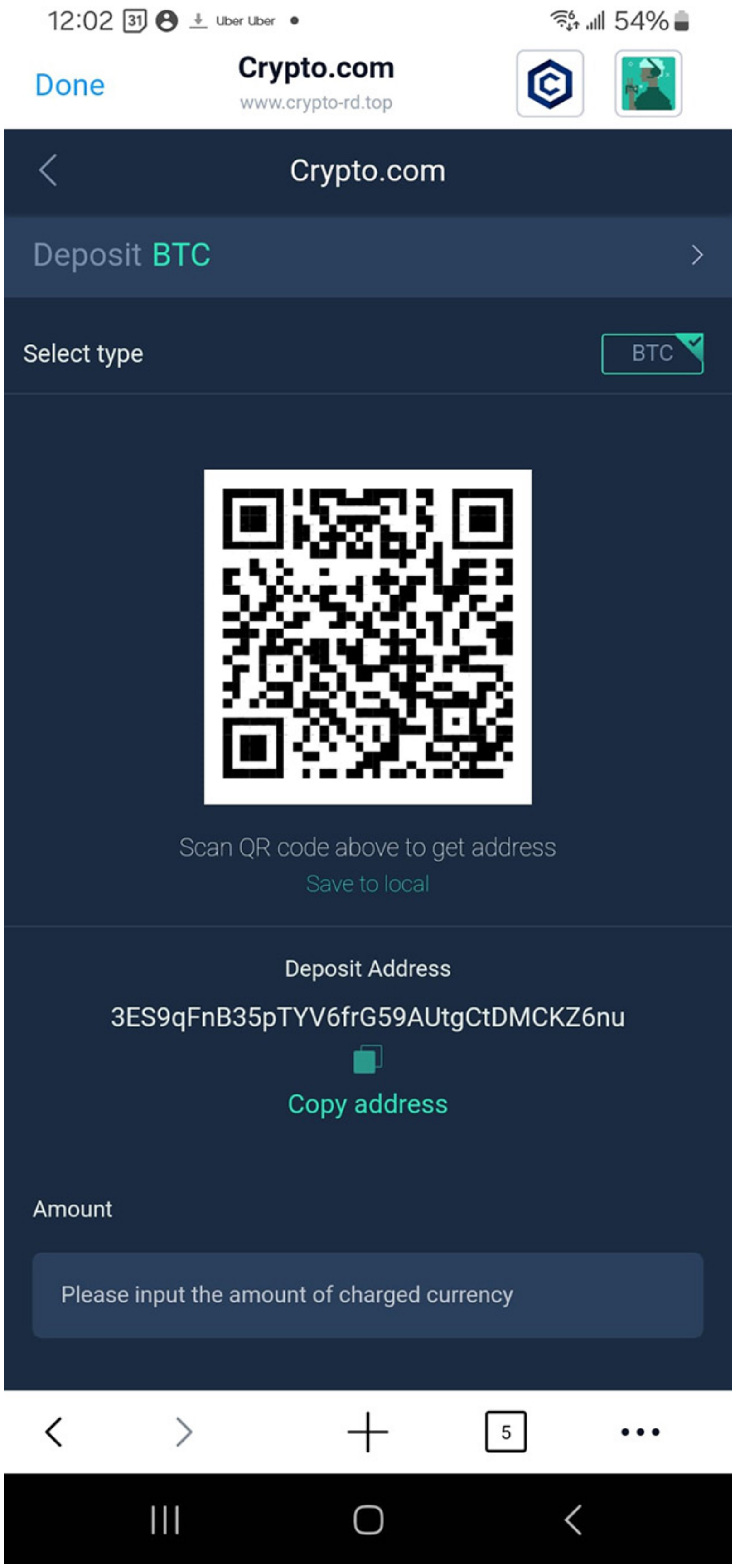

FIGURE S8. Screenshot evidence for Case #66.

Source: DFPI Crypto Scam Tracker (public). File: pb_023_case_066.jpg

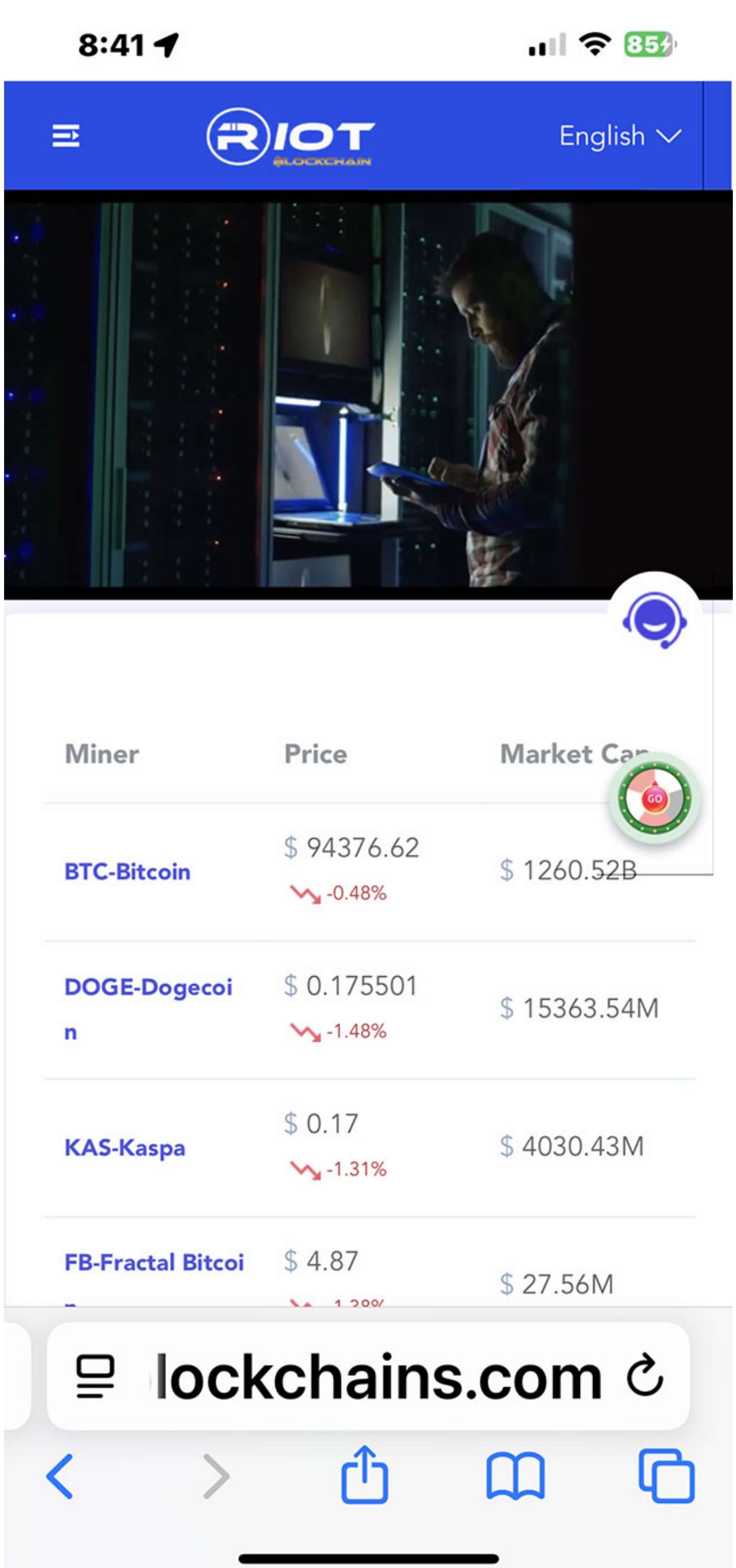

FIGURE S9. Screenshot evidence for Case #67.

Source: DFPI Crypto Scam Tracker (public). File: pb_024_case_067.jpg

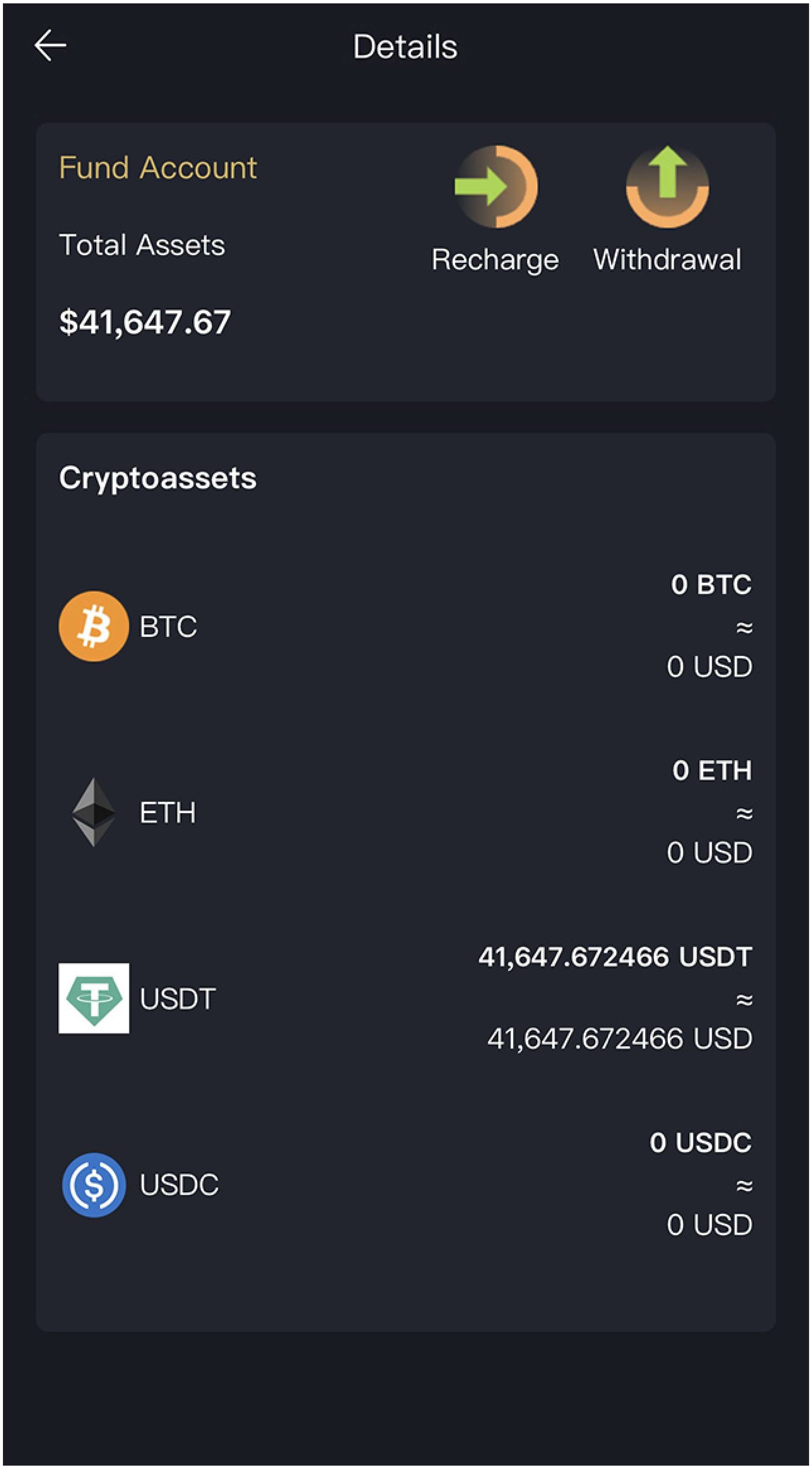

FIGURE S10. Screenshot evidence for Case #68.

Source: DFPI Crypto Scam Tracker (public). File: pb_025_case_068.jpg

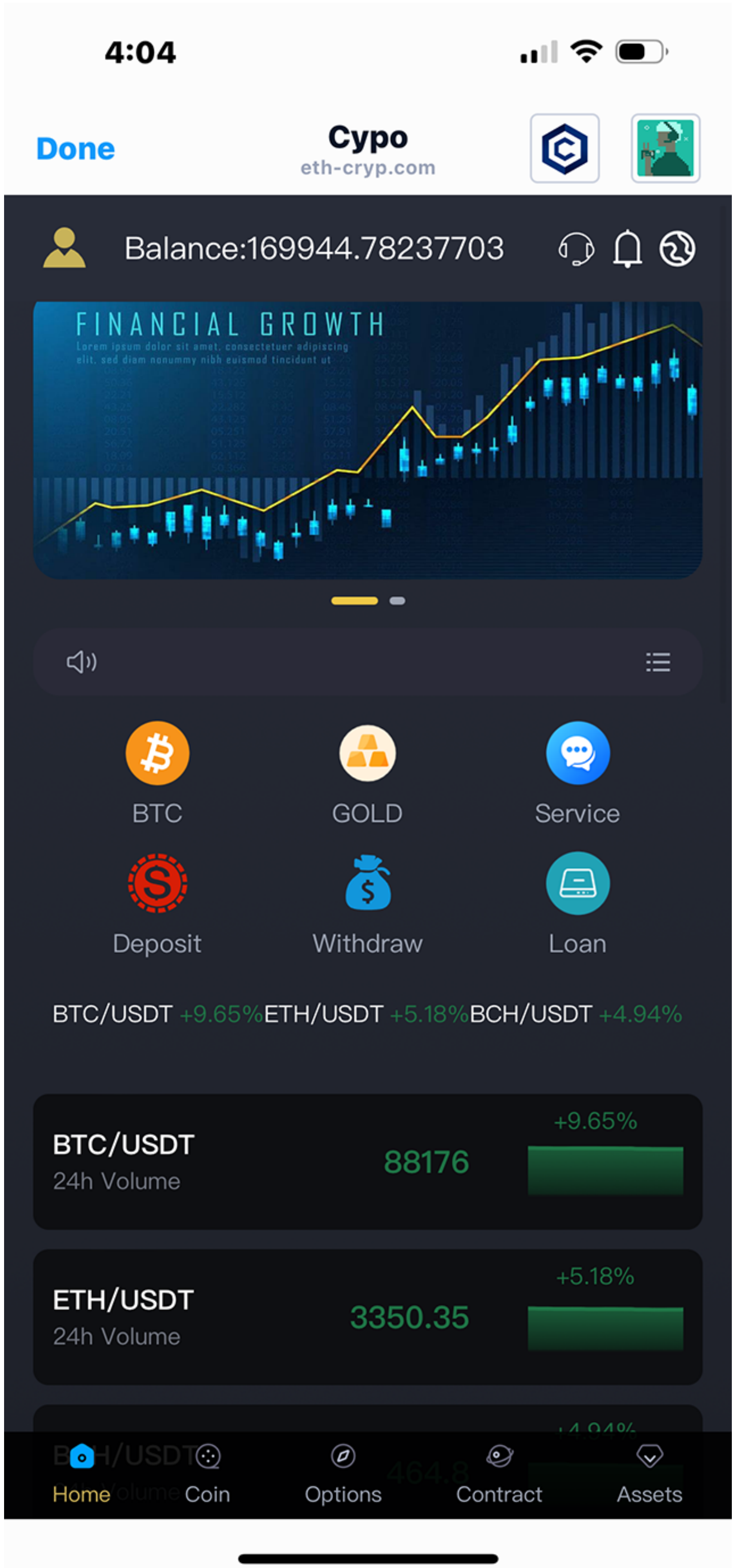

FIGURE S11. Screenshot evidence for Case #69.

Source: DFPI Crypto Scam Tracker (public). File: pb_026_case_069.png

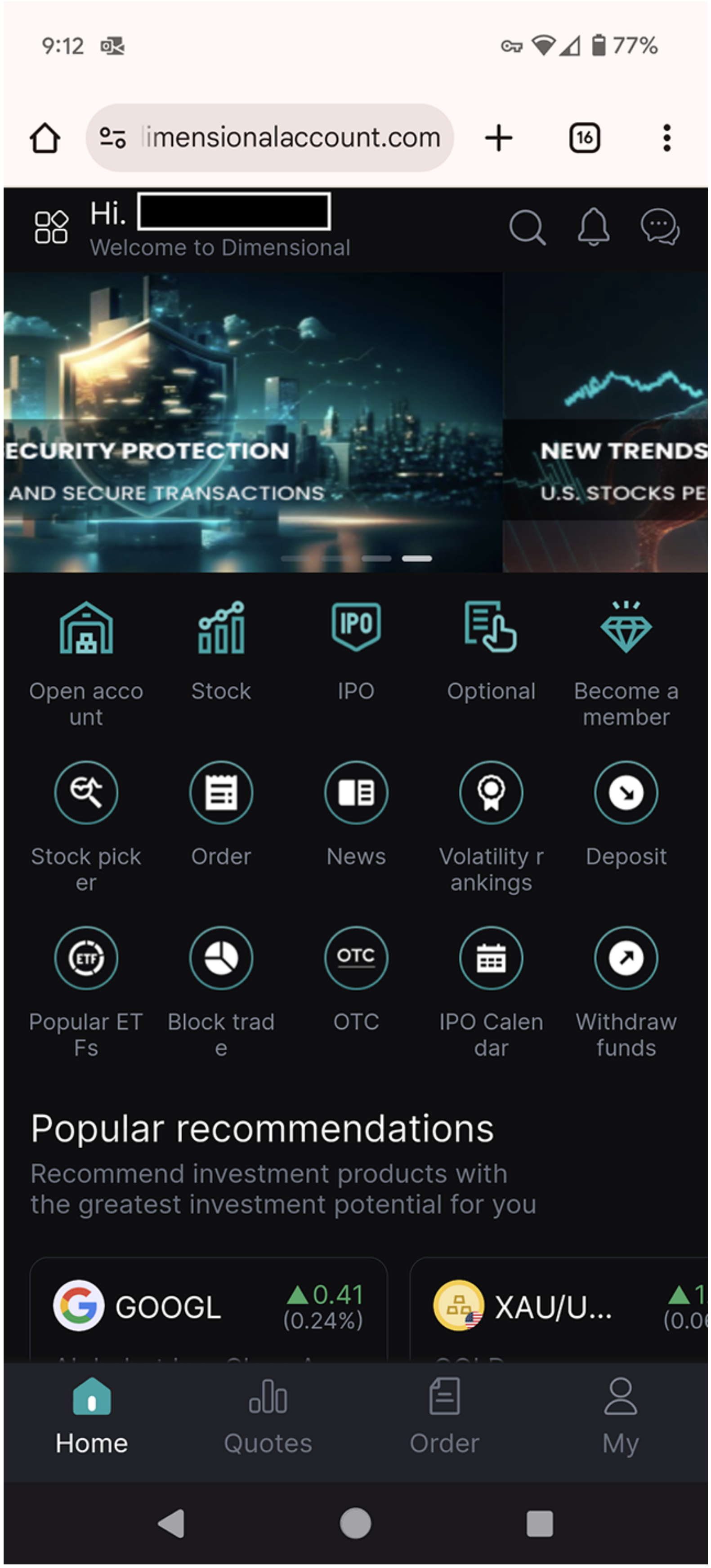

FIGURE S12. Screenshot evidence for Case #91.

Source: DFPI Crypto Scam Tracker (public). File: pb_032_case_091.png

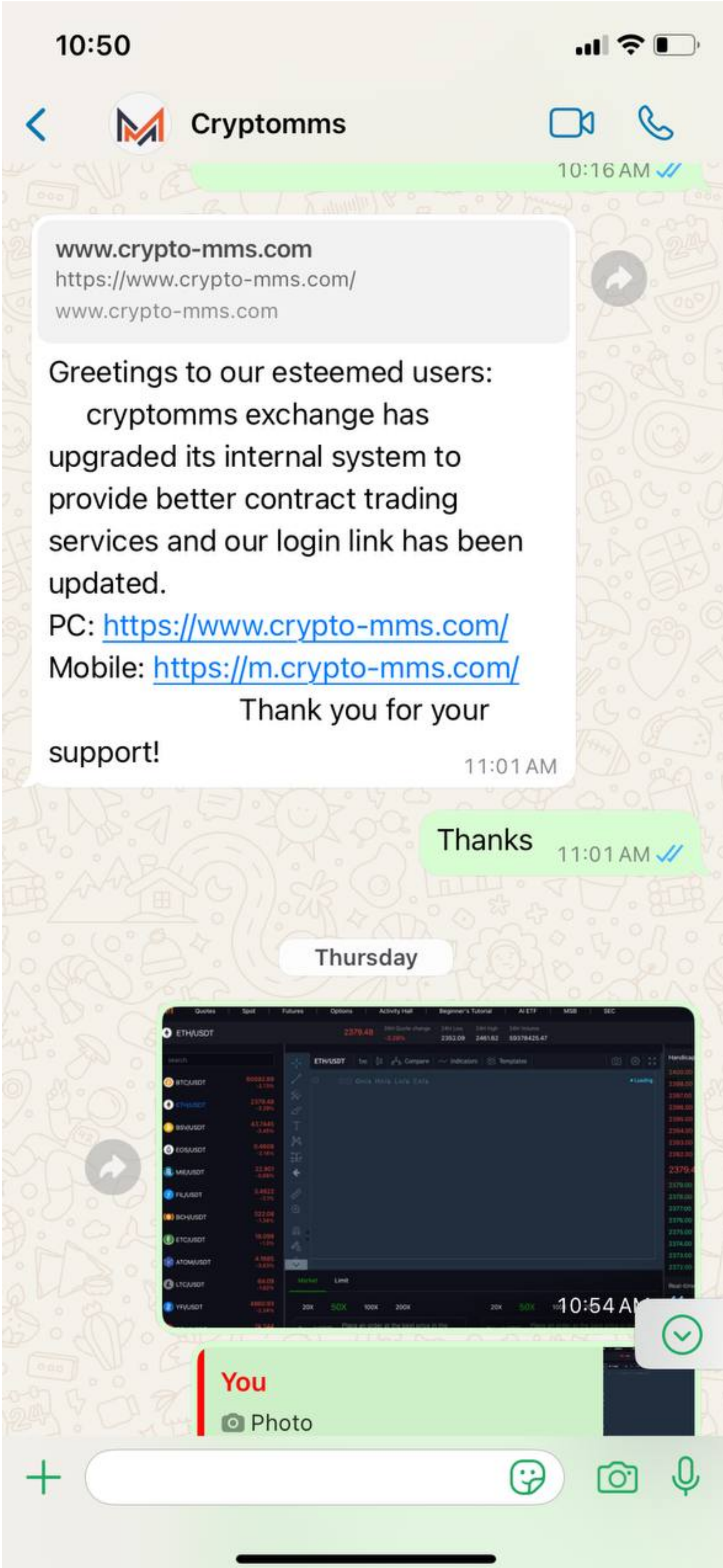

FIGURE S13. Screenshot evidence for Case #107.

Source: DFPI Crypto Scam Tracker (public). File: pb_036_case_107.jpeg

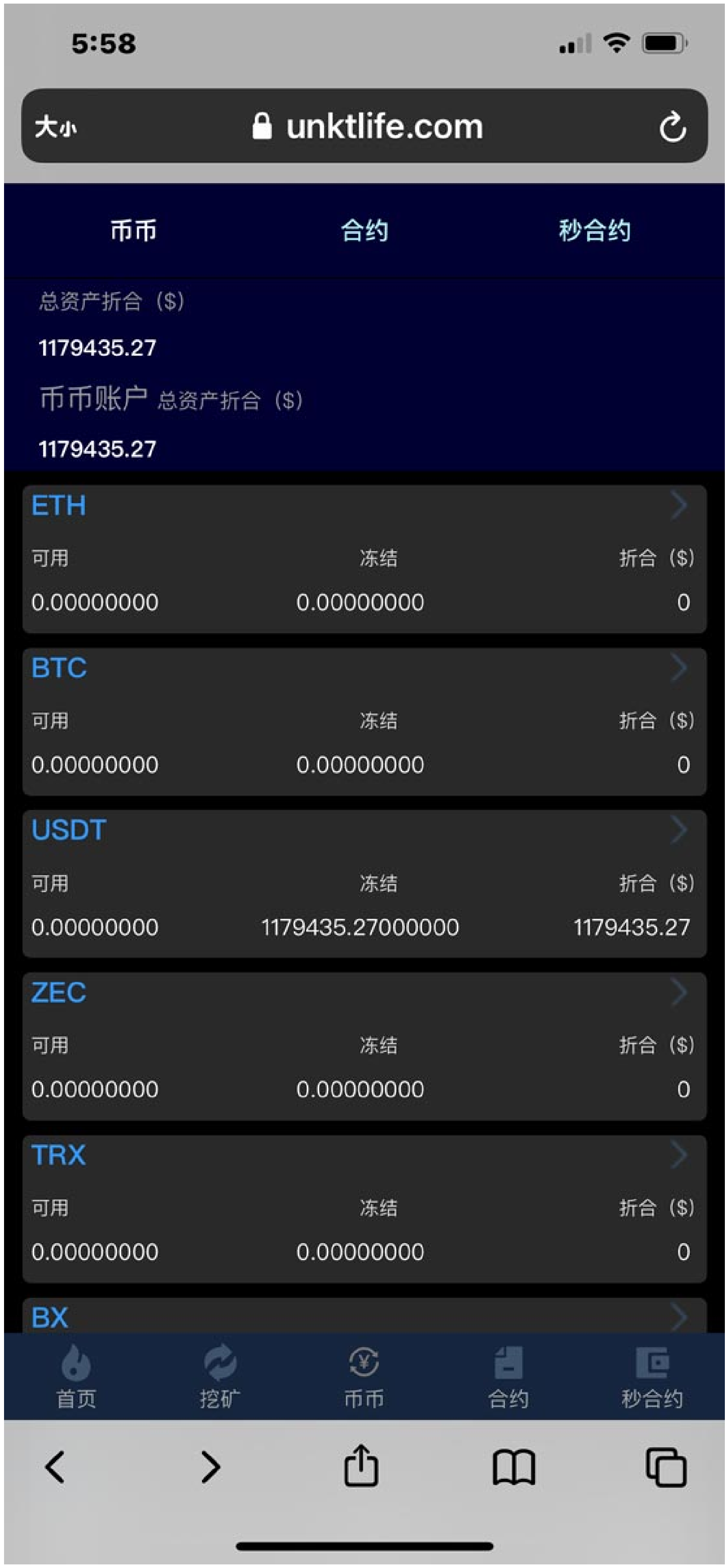

FIGURE S14. Screenshot evidence for Case #111.

Source: DFPI Crypto Scam Tracker (public). File: pb_037_case_111.jpg

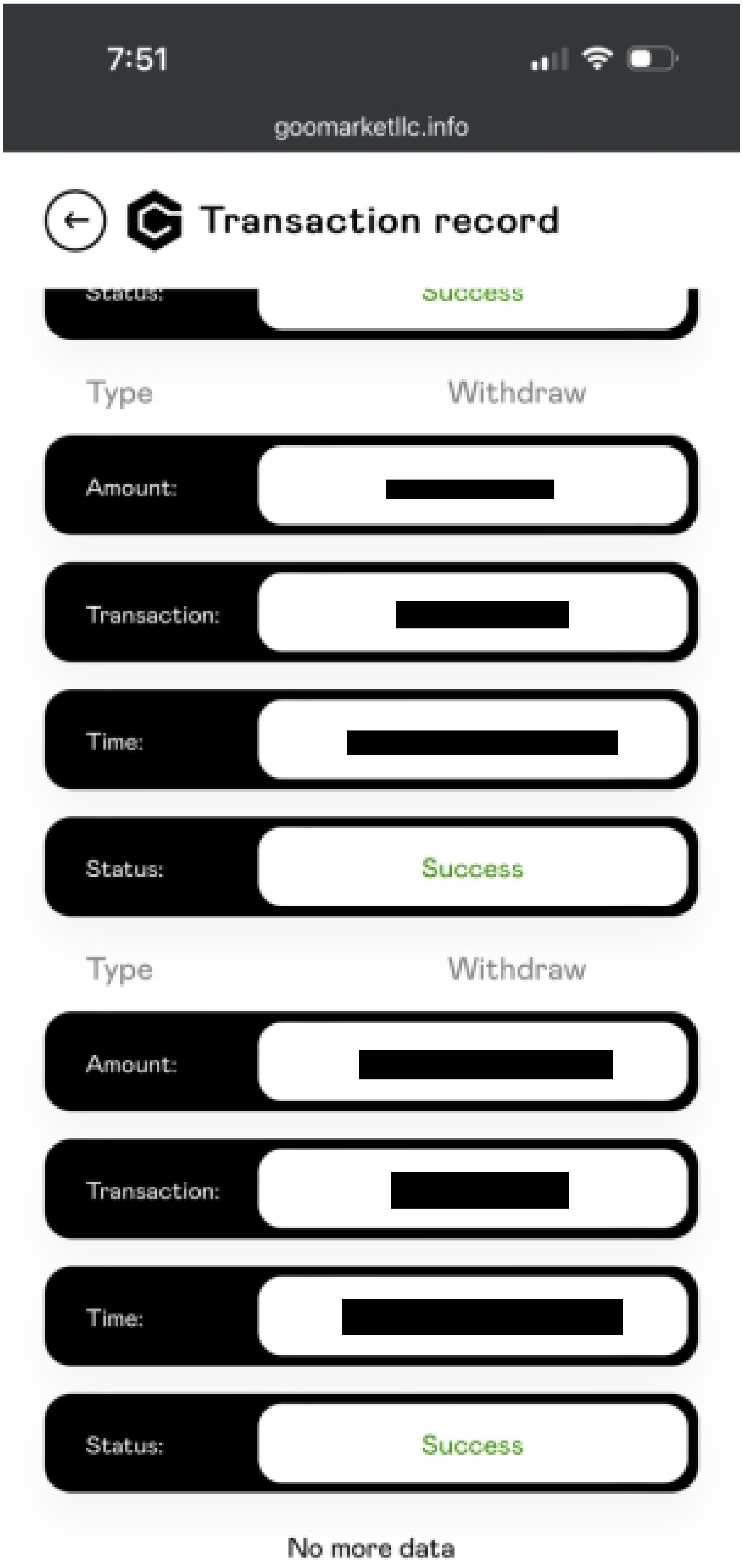

FIGURE S15. Screenshot evidence for Case #120.

Source: DFPI Crypto Scam Tracker (public). File: pb_042_case_120.png

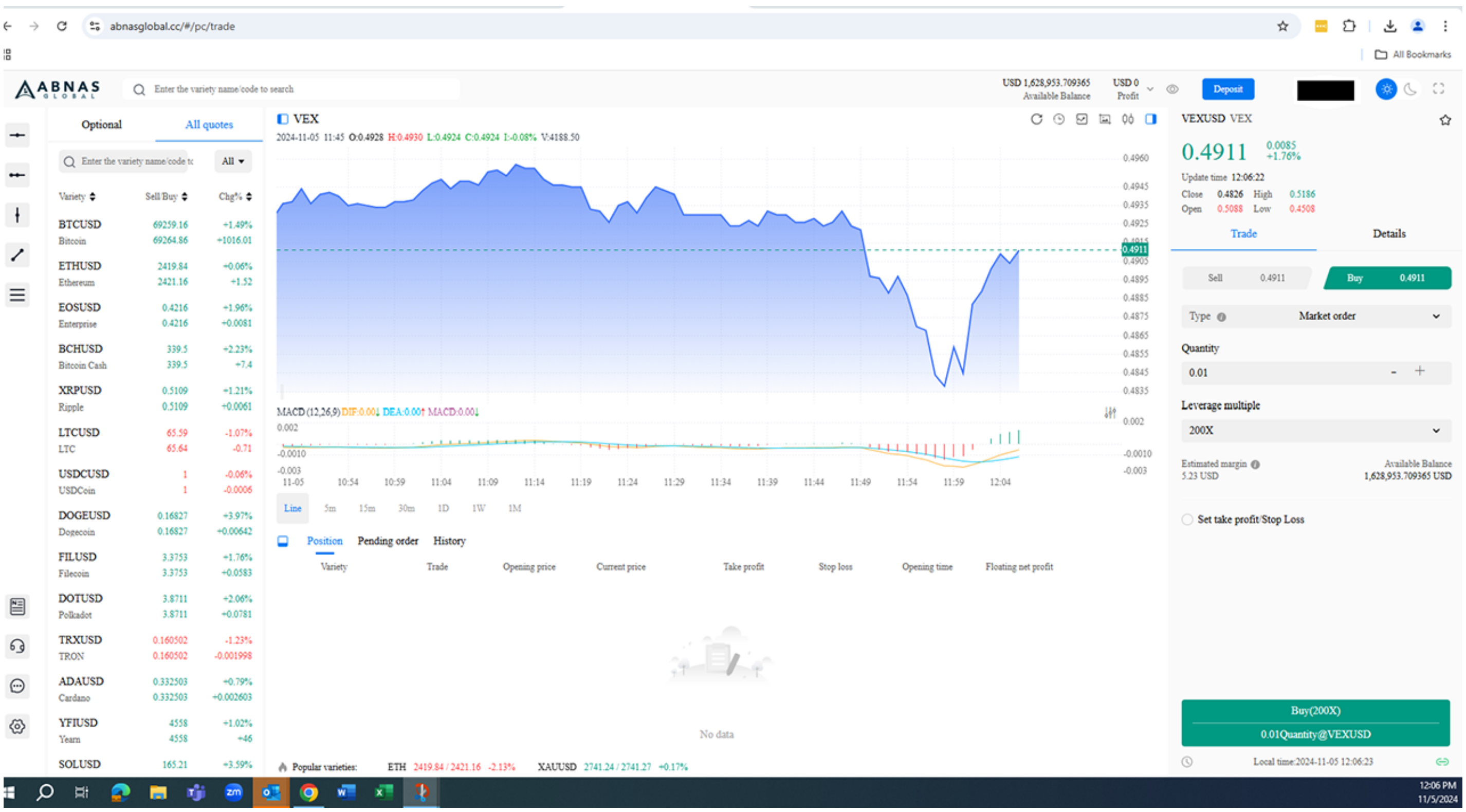

FIGURE S16. Screenshot evidence for Case #135.

Source: DFPI Crypto Scam Tracker (public). File: pb_046_case_135.png

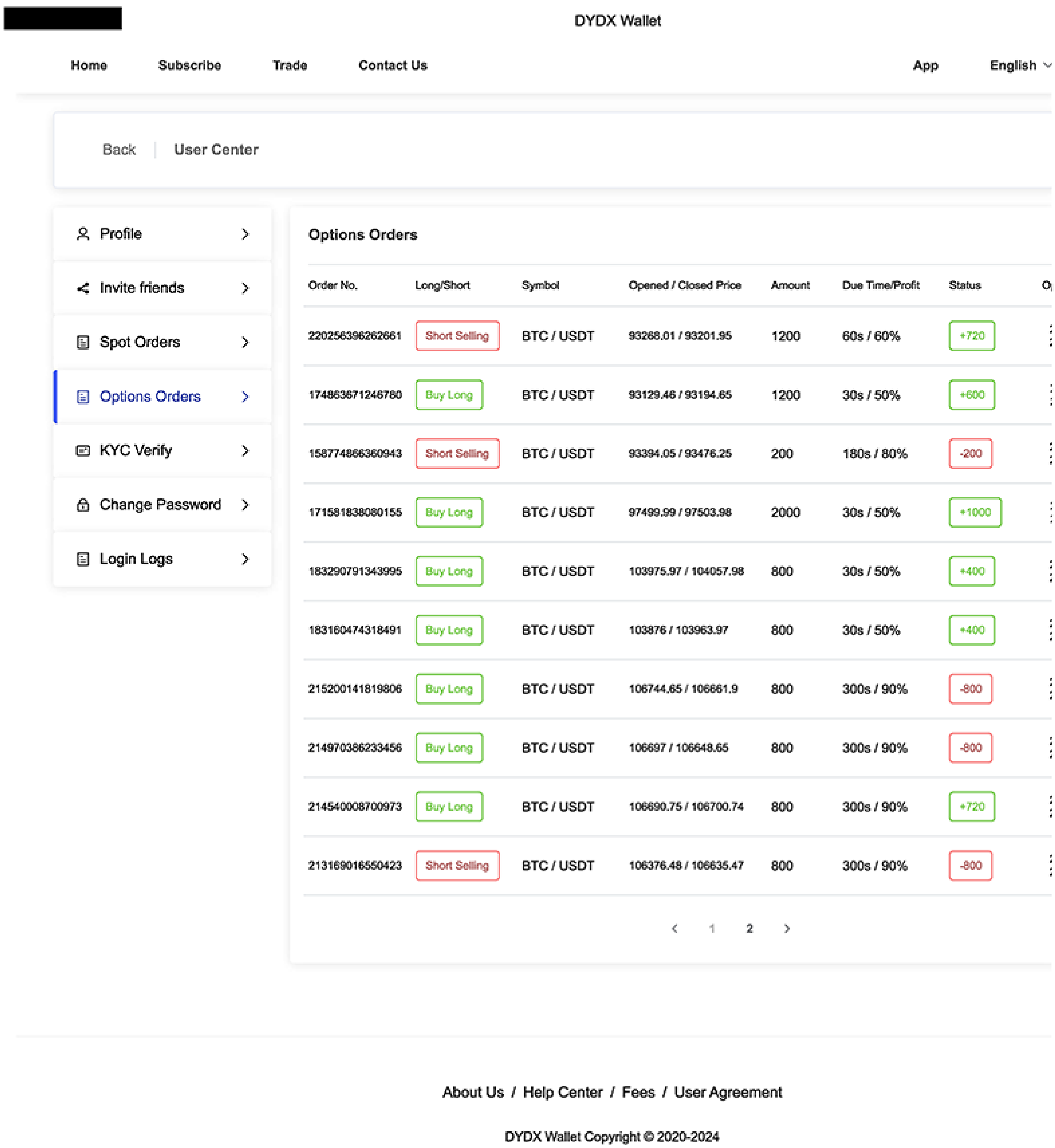
[redacted]

DYDX Wallet

Home Subscribe Trade Contact Us App English

Back | User Center

Profile
Invite friends
Spot Orders
Options Orders
KYC Verify
Change Password
Login Logs

Options Orders

Order No.	Long/Short	Symbol	Opened / Closed Price	Amount	Due Time/Profit	Status	O
220256396262661	Short Selling	BTC / USDT	93268.01 / 93201.95	1200	60s / 60%	+720	
174863671246780	Buy Long	BTC / USDT	93129.46 / 93194.65	1200	30s / 50%	+600	
158774866360943	Short Selling	BTC / USDT	93394.05 / 93476.25	200	180s / 80%	-200	
171581838080155	Buy Long	BTC / USDT	97499.99 / 97503.98	2000	30s / 50%	+1000	
183290791343995	Buy Long	BTC / USDT	103975.97 / 104057.98	800	30s / 50%	+400	
183160474318491	Buy Long	BTC / USDT	103876 / 103963.97	800	30s / 50%	+400	
215200141819806	Buy Long	BTC / USDT	106744.65 / 106661.9	800	300s / 90%	-800	
214970386233456	Buy Long	BTC / USDT	106697 / 106648.65	800	300s / 90%	-800	
214540008700973	Buy Long	BTC / USDT	106690.75 / 106700.74	800	300s / 90%	+720	
213169016550423	Short Selling	BTC / USDT	106376.48 / 106635.47	800	300s / 90%	-800	

‹ 1 2 ›

About Us / Help Center / Fees / User Agreement

DYDX Wallet Copyright © 2020-2024

FIGURE S17. Screenshot evidence for Case #136.

Source: DFPI Crypto Scam Tracker (public). File: pb_047_case_136.png

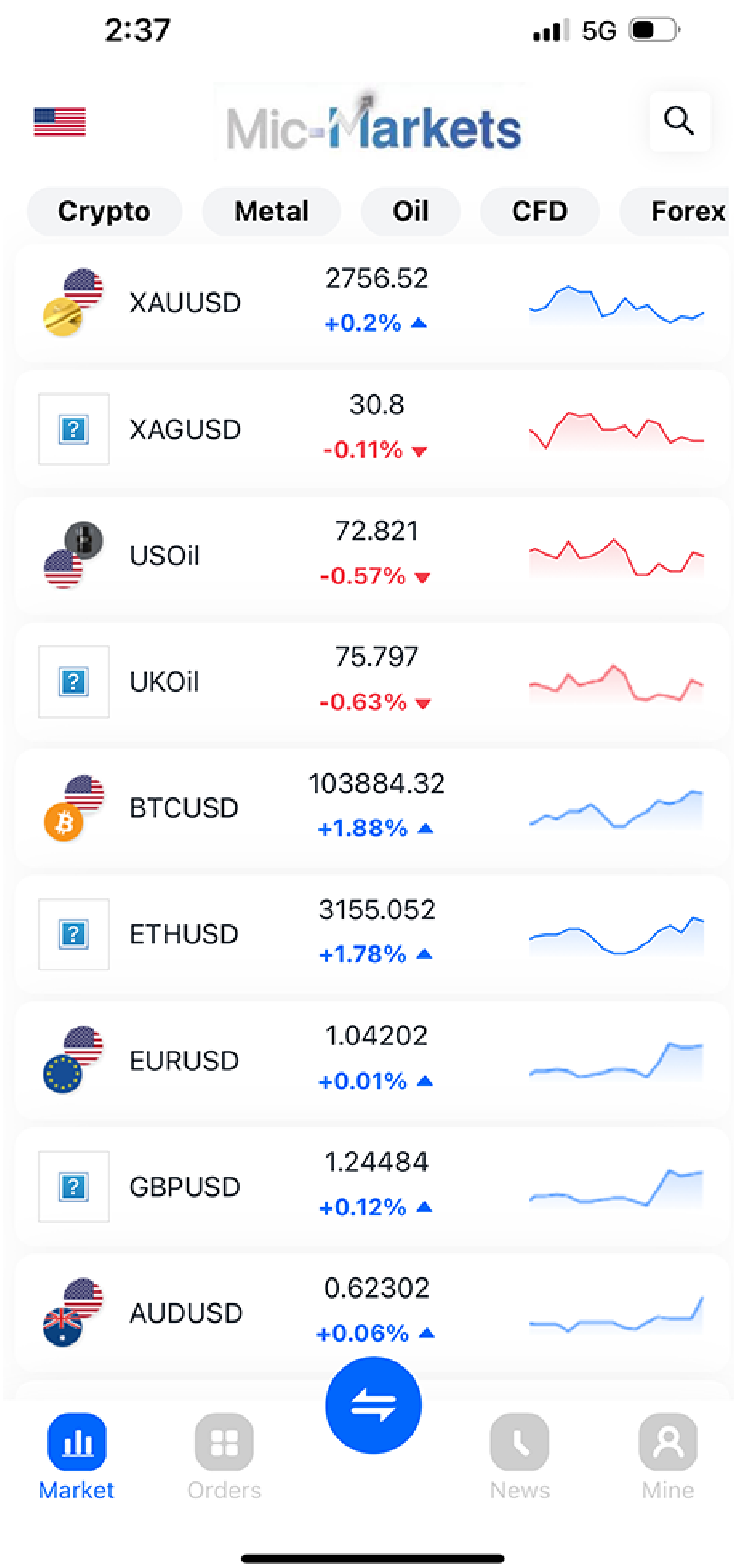

FIGURE S18. Screenshot evidence for Case #137.

Source: DFPI Crypto Scam Tracker (public). File: pb_048_case_137.png

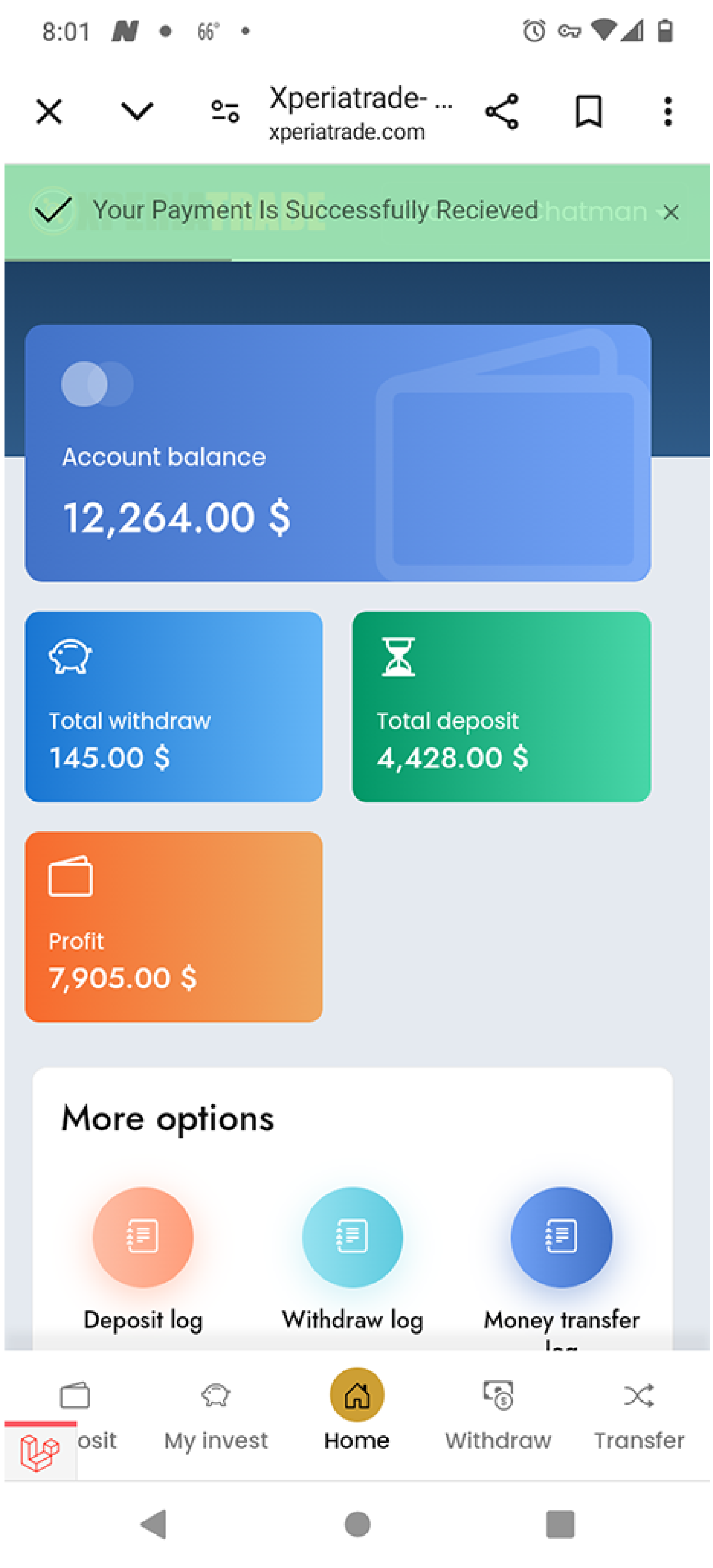

FIGURE S19. Screenshot evidence for Case #139.

Source: DFPI Crypto Scam Tracker (public). File: pb_049_case_139.png

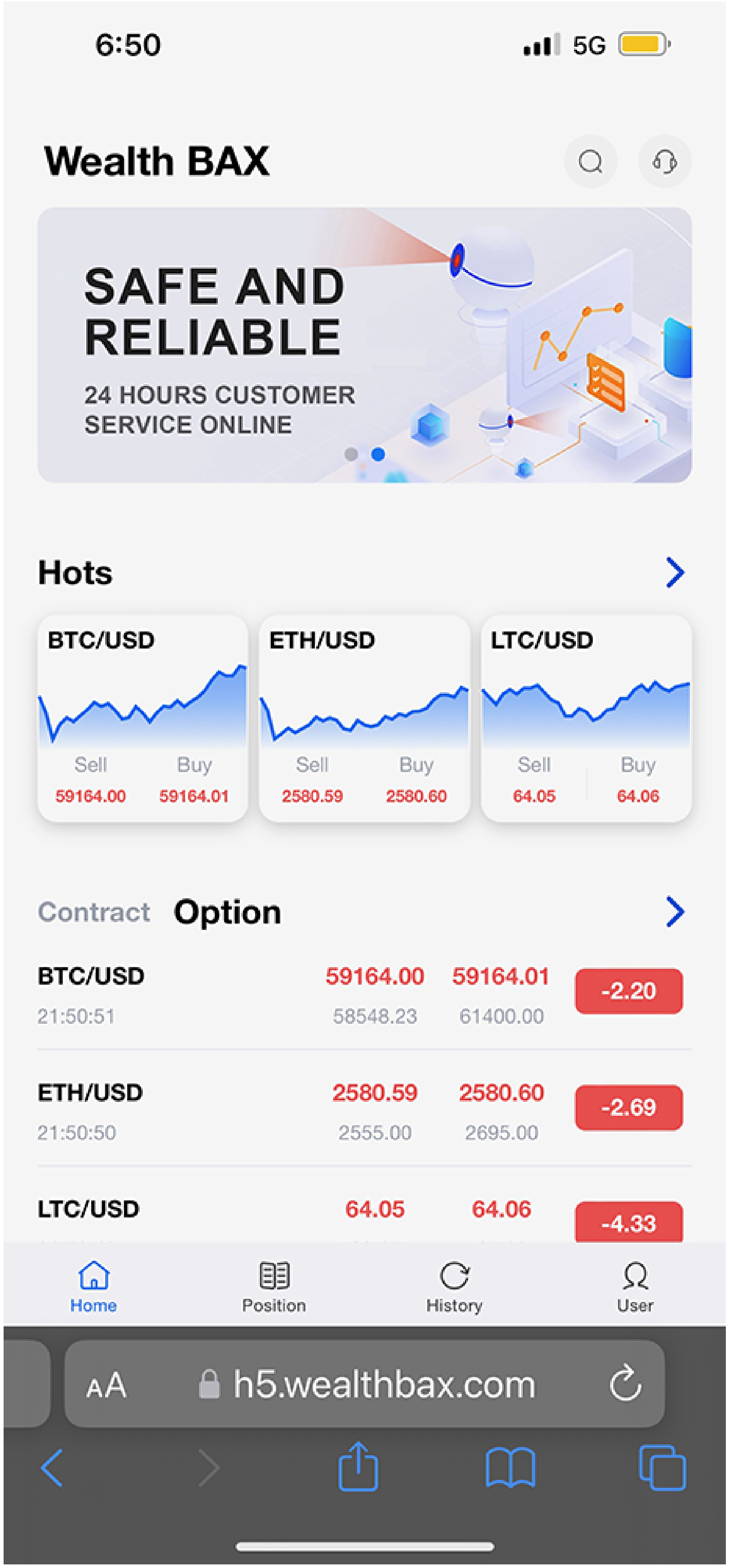

FIGURE S20. Screenshot evidence for Case #158.

Source: DFPI Crypto Scam Tracker (public). File: pb_057_case_158.jpg

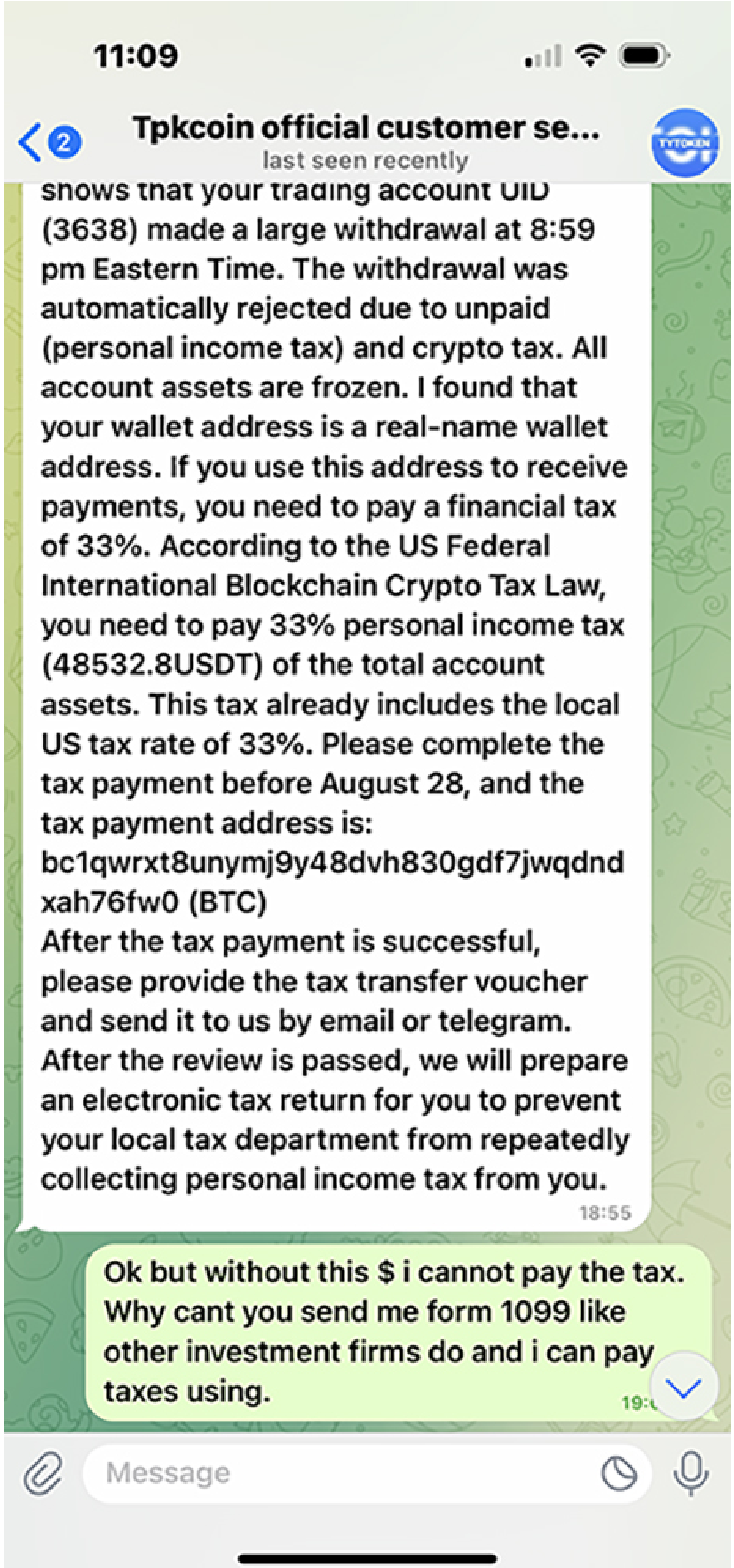

FIGURE S21. Screenshot evidence for Case #159.

Source: DFPI Crypto Scam Tracker (public). File: pb_058_case_159.jpg

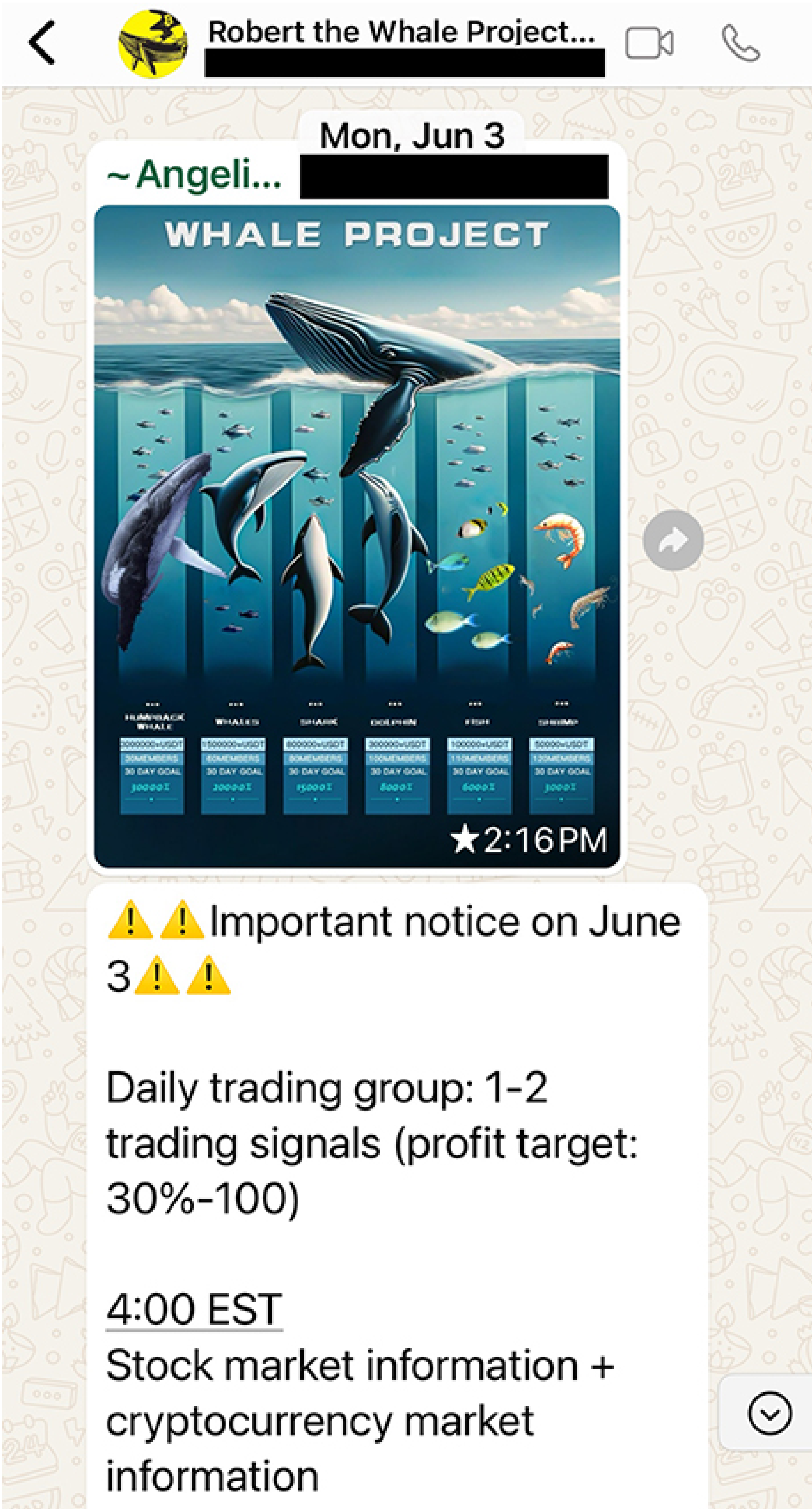

FIGURE S22. Screenshot evidence for Case #160.

Source: DFPI Crypto Scam Tracker (public). File: pb_059_case_160.jpg

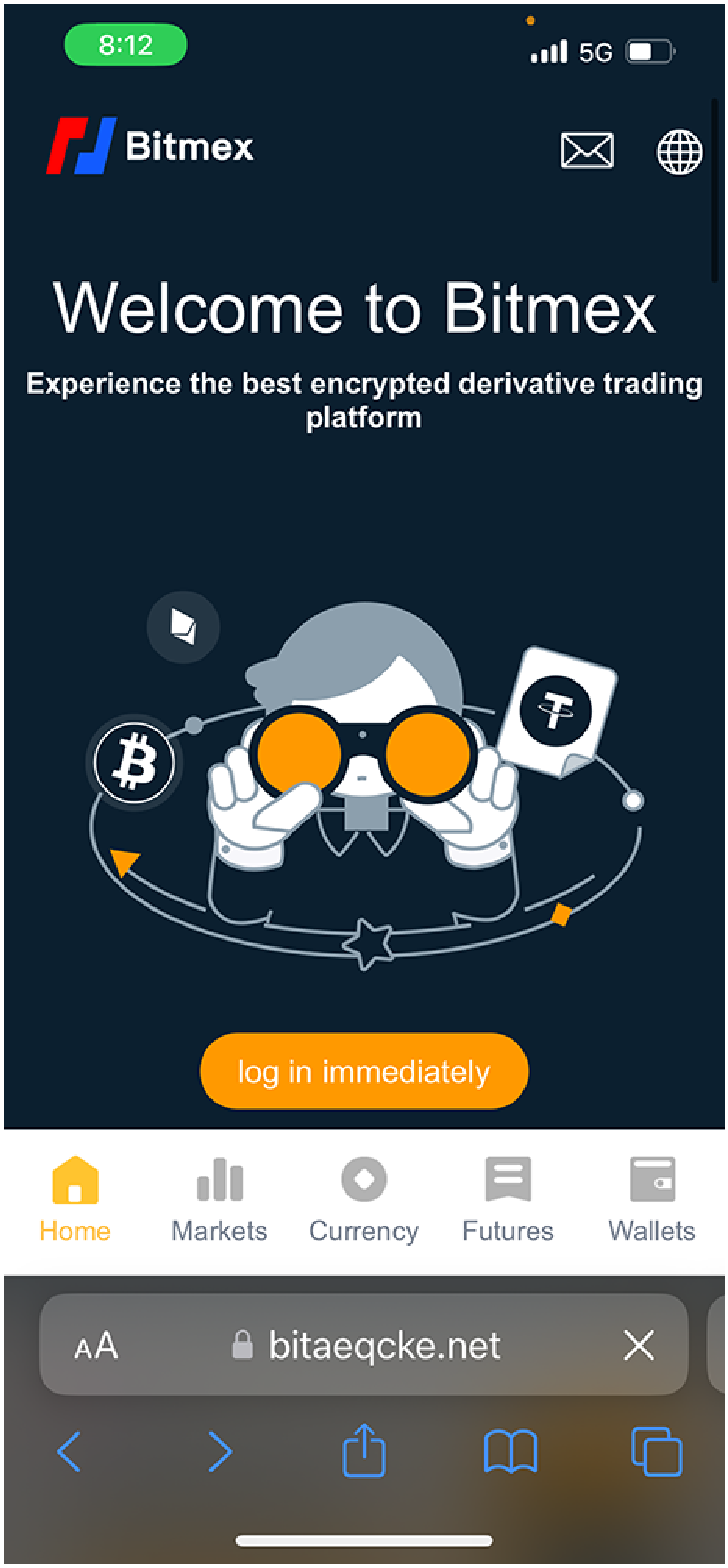

FIGURE S23. Screenshot evidence for Case #161.

Source: DFPI Crypto Scam Tracker (public). File: pb_060_case_161.png

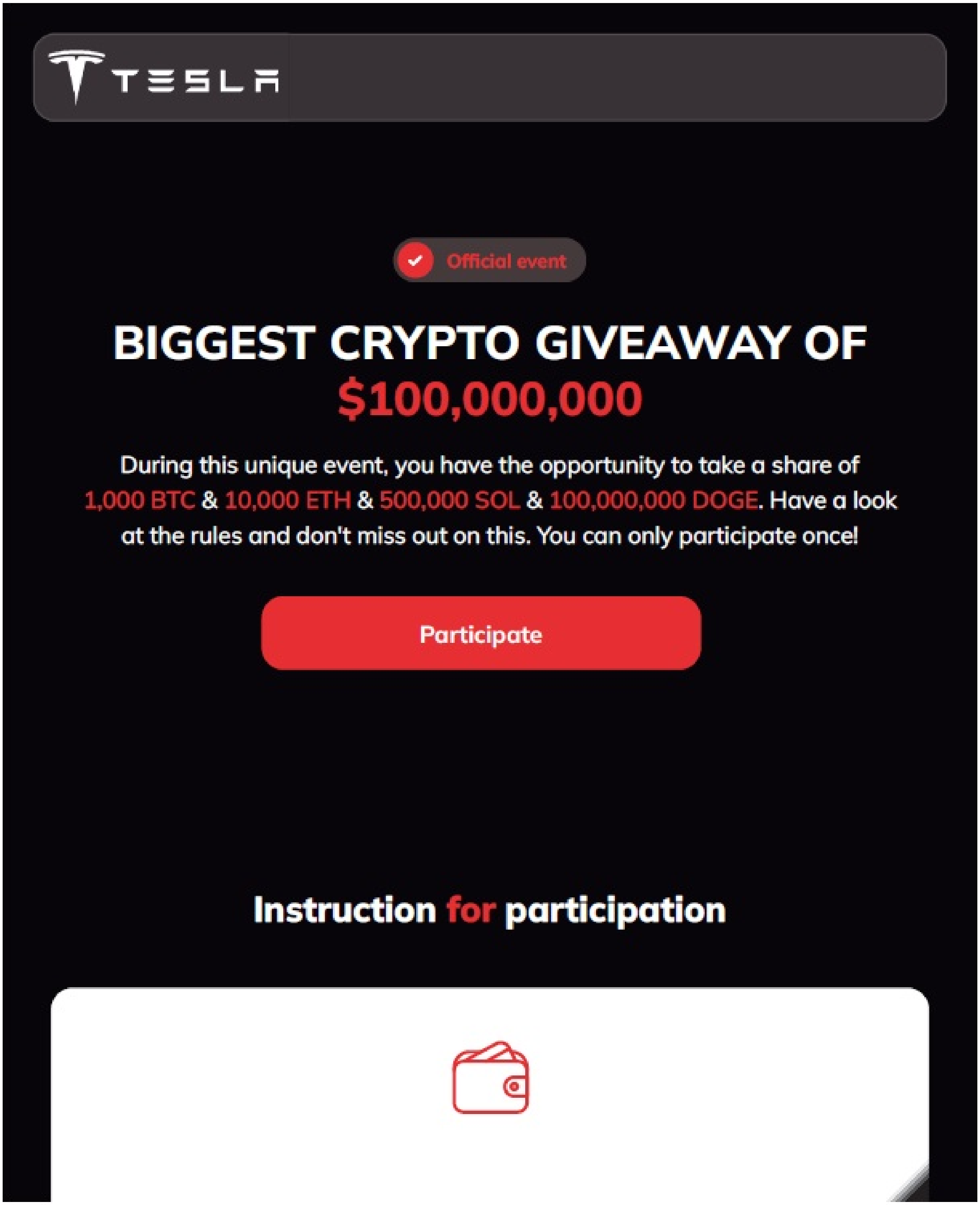

https://webelon.org/

8/30/24, 1:46 PM
Page 1 of 7

FIGURE S24. Screenshot evidence for Case #162.

Source: DFPI Crypto Scam Tracker (public). File: pb_061_case_162.jpg

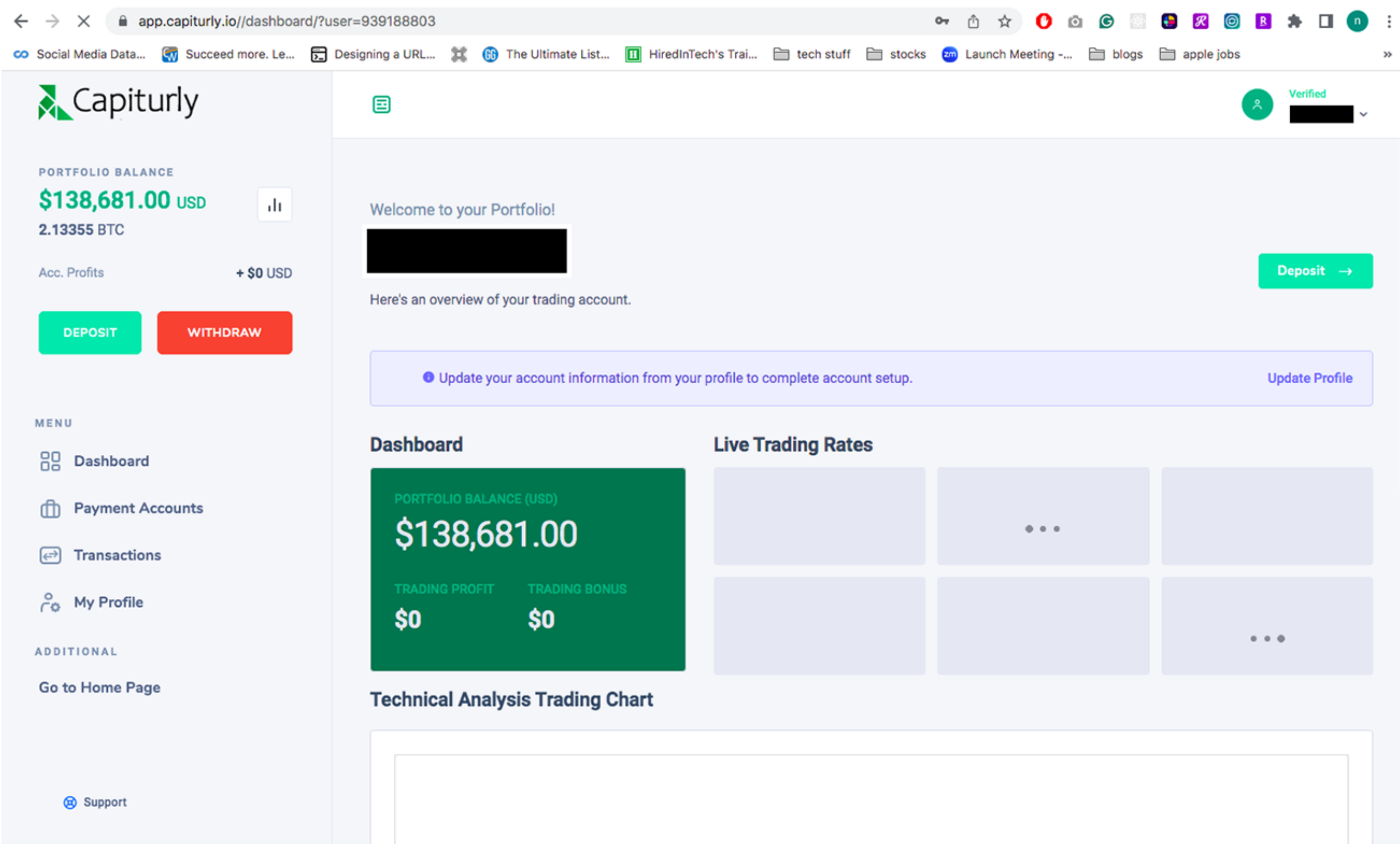

FIGURE S25. Screenshot evidence for Case #163.

Source: DFPI Crypto Scam Tracker (public). File: pb_062_case_163.png
